# Guided Antitumoural Drugs: (Imidazol‐2‐ylidene)(L)gold(I) Complexes Seeking Cellular Targets Controlled by the Nature of Ligand L

**DOI:** 10.1002/chem.202005451

**Published:** 2021-02-08

**Authors:** Sofia I. Bär, Madeleine Gold, Sebastian W. Schleser, Tobias Rehm, Alexander Bär, Leonhard Köhler, Lucas R. Carnell, Bernhard Biersack, Rainer Schobert

**Affiliations:** ^1^ Organic Chemistry Laboratory University Bayreuth Universitaetsstr. 30 95447 Bayreuth Germany

**Keywords:** cancer, drug discovery, gold, metallodrugs, subcellular localisation

## Abstract

Three [1,3‐diethyl‐4‐(*p*‐methoxyphenyl)‐5‐(3,4,5‐trimethoxyphenyl)imidazol‐2‐ylidene](L)gold(I) complexes, **4 a** (L=Cl), **5 a** (L=PPh_3_), and **6 a** (L=same N‐heterocyclic carbene (NHC)), and their fluorescent [4‐(anthracen‐9‐yl)‐1,3‐diethyl‐5‐phenylimidazol‐2‐ylidene](L)gold(I) analogues, **4 b**, **5 b**, and **6 b**, respectively, were studied for their localisation and effects in cancer cells. Despite their identical NHC ligands, the last three accumulated in different compartments of melanoma cells, namely, the nucleus (**4 b**), mitochondria (**5 b**), or lysosomes (**6 b**). Ligand L was also more decisive for the site of accumulation than the NHC ligand because the couples **4 a**/**4 b**, **5 a**/**5 b**, and **6 a**/**6 b**, carrying different NHC ligands, afforded similar results in cytotoxicity tests, and tests on targets typically found at their sites of accumulation, such as DNA in nuclei, reactive oxygen species and thioredoxin reductase in mitochondria, and lysosomal membranes. Regardless of the site of accumulation, cancer cell apoptosis was eventually induced. The concept of guiding a bioactive complex fragment to a particular subcellular target by secondary ligand L could reduce unwanted side effects.

## Graphical abstract

**Striking gold**: Through the choice of their secondary ligand L, a series of [4,5‐diarylimidazol‐2‐ylidene](L)gold(I) complexes targeted different cancer cell organelles (nuclei, mitochondria or lysosomes) where they interfered beneficially with molecular targets, such as DNA, thioredoxin reductase, or lysosomal membranes, to elicit p53‐independent apoptosis. Such guided metallodrugs will help to minimise unwanted side effects by addressing limited arrays of targets.

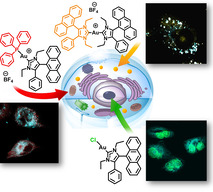

## Introduction

**Scheme 1:**
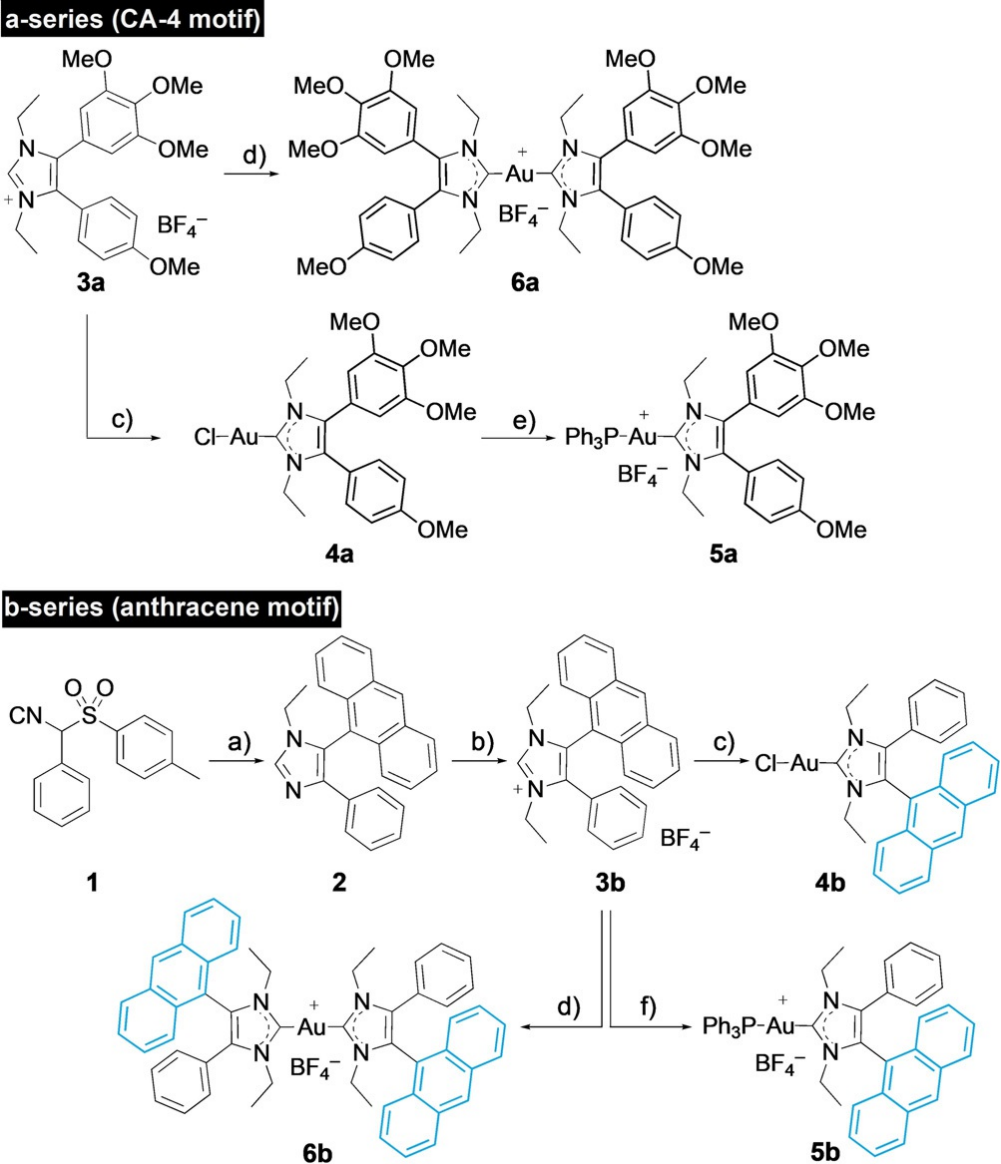
Syntheses of complexes **4**–**6**: a) 9‐formylanthracene, EtNH_2_/THF, AcOH, EtOH, reflux, 2 h, then **1**, K_2_CO_3_, reflux 6 h, 61 %; b) 1) EtI, MeCN, reflux, 48 h; 2) NaBF_4_, acetone, RT, 1 h, 95 %; c) Ag_2_O (0.5 equiv), CH_2_Cl_2_, RT, 5 h, then [AuCl(SMe_2_)] (1 equiv), LiCl, RT, 24 h, 92 %; d) Ag_2_O (0.5 equiv), CH_2_Cl_2_, RT, 5 h, then [AuCl(SMe_2_)] (0.5 equiv), RT, 24 h, 88 %; e) PPh_3_, NaBF_4_, CH_2_Cl_2_, RT, 24 h, 79 %; f) [AuCl(PPh_3_)], KO*t*Bu, CH_2_Cl_2_, RT, 24 h, 70 %.

Although N‐heterocyclic carbene (NHC) complexes have been much used as catalysts, their medicinal relevance was recognised surprisingly late, given their chemical stability under physiological conditions and their structural flexibility.[^1^, ^2^] Unlike cisplatin (CDDP) and related platinum coordination complexes, which all lead to DNA adducts, resulting in an inhibition of the cancer cell cycle and eventually in apoptotic cancer cell death,^[3]^ NHC complexes of various metals may address a broader array of molecular targets. Complexes with the character of delocalised lipophilic cations (DLCs) were found to selectively accumulate in mitochondria, which can be explained by their negative inner transmembrane potential.[^4^, ^5^] Because cancer cells have a more hyperpolarised mitochondrial membrane potential (MMP) than normal cells, the selective accumulation of metal–carbene complexes with DLC character in cancer cells can be expected.[^5^, ^6^] With the detection of antitumour activity of the antirheumatic gold(I) compound auranofin, (2,3,4,6‐tetra‐*O*‐acetyl‐1‐thio‐β‐d‐glucopyranosato)(triethylphosphane)gold, gold complexes came to the fore as potential anticancer drug candidates.^[7]^ Auranofin mainly acts through the inhibition of mitochondrial thioredoxin reductase (TrxR) and by enhancing the mitochondrial permeability.[^8^, ^9^] Through the inhibition of TrxR activity, the intracellular levels of reactive oxygen species (ROS) rise, which damages predominantly cancer cells because of their elevated ROS levels compared with healthy cells.^[10]^ As a result, cytochrome c is released into the cytosol, triggering apoptotic cell death.^[11]^ Due to their stability, NHC ligands can also be annulated and substituted in multifarious ways, allowing the mimicking or combinatorial attachment of pharmacophores to afford pleiotropic drugs.^[12]^ Herein, we report on NHC gold(I) complexes **4 a**–**6 a**, carrying a 1,3‐diethyl‐4‐(4‐methoxyphenyl)‐5‐(3,4,5‐trimethoxyphenyl)imidazol‐2‐ylidene ligand, akin to the natural antimitotic combretastatin A4 (CA‐4), and differing only in the second ligand on the gold atom (Scheme 1). Preliminary studies had shown strong cytotoxicity against cancer cells with IC_50_ values in the low triple‐ to double‐digit nanomolar range for complex **6 a**, but its actual mechanism of action remained unclear.^[13]^ A second series of complexes **4 b**–**6 b**, bearing the same “second ligands L”, yet a better detectable fluorescent 1,3‐diethyl‐4‐(anthracen‐9‐yl)‐5‐phenylimidazol‐2‐ylidene ligand, were synthesised and studied for their intracellular accumulation and their modes of anticancer action. The aim of this study was to find out whether ligand L could be used to set the site of accumulation, and thus, the targets and nature of antitumour effects of gold complexes with identical or closely related NHC ligands. This was particularly tempting because similar *cis*‐[{bis(1,3‐dibenzylimidazol‐2‐ylidene)Cl(L)}Pt^II^] complexes were previously shown by us to always accumulate in mitochondria, regardless of the charge of the complex and nature of ligands L.^[14]^ Likewise, Ott et al. reported a triad of (1,3‐diethylbenzimidazol‐2‐ylidene)(L)gold(I) complexes with the same ligands (L=Cl, PPh_3_, NHC), which all localised in the mitochondria, albeit to different degrees.^[15]^

## Results and Discussion

### Synthesis

The new gold(I) NHC complexes were prepared from imidazolium salts **3 a** and **3 b** (Scheme 1). Compound **3 b** was synthesised analogously to known compound **3 a** by the van Leusen reaction of toluenesulfonylmethyl isocyanide (TosMIC) reagent **1** with 9‐formylanthracene, followed by N‐alkylation and anion exchange of the resulting imidazole **2**. Reactions of **3 a** and **3 b** with Ag_2_O and transmetalation of the corresponding silver carbene complexes with different amounts of [AuCl(SMe_2_)] afforded mono‐ and bis‐carbene gold(I) complexes **4 a/b** and **6 a/b** analogously to literature procedures.[^12^, ^13^] New cationic complex **5 a** was prepared by the reaction of complex **4 a** with triphenylphosphane. Complex **5 b** was obtained by diprotonation of **3 b** and reaction of the free carbene with [AuCl(PPh_3_)]. The stability of all complexes **4**–**6** in aqueous solution was ascertained by ^1^H NMR spectroscopic monitoring over a period of 72 h (see the Supporting Information).

### Cytotoxicity against cancer cells

All complexes **4**–**6** had an antiproliferative effect, with IC_50_ values in the three‐digit nanomolar to low double‐digit micromolar range, on cells of the human cancer cell lines HCT‐116^wt^, its p53 knockout mutant HCT‐116^p53−/−^ (both colon cancer), 518A2 (melanoma), HeLa, and multi‐drug‐resistant KB‐V1^Vbl^ (both cervical carcinoma; Table 1). For complexes **4 a**, **5 a** and **6 a** bearing a CA‐4 analogous NHC ligand, we found that the cytotoxicity increased with their DLC character, that is, in the order **4 a**<**5 a**<**6 a**, except for the KB‐V1^Vbl^ cells. A similar trend was observed for the anthracenyl complexes (**4 b<5 b<6 b**), with the exception of bis‐NHC complex **6 b**, which is less active than phosphane complex **5 b** in 518A2 melanoma and HeLa cervical carcinoma cells. This conformity of cytotoxicities of the **a** and **b** series of complexes suggests similar mechanisms of action. Interestingly, all tested gold complexes, including auranofin, were more active against the p53‐knockout mutant HCT‐116^p53−/−^, if compared with its wild‐type analogue HCT‐116^wt^ expressing functional p53 protein. We assume that complexes **4**–**6** induce cancer cell death in a way that is independent of p53, as already shown for auranofin[^16^, ^17^] and for related (1,3‐diethylbenzimidazol‐2‐ylidene)gold(I) complexes.^[18]^ Complexes **4 b**, **5** and **6** were also quite active against the multi‐drug‐resistant cell line KB‐V1^Vbl^, which expresses high levels of Pg‐p, an ATP‐dependent efflux pump, capable of expelling a variety of xenobiotics. Complexes **5 a** and **6 b** appear to have a particularly low affinity for Pg‐p. Cationic complexes **5 b** and **6 a** showed some selectivity for cancer over non‐malignant cells and are particularly interesting candidates for further studies.

**Table 1:** Inhibitory concentrations, IC_50_^[a]^ [μm], of complexes **4**–**6** upon application to cells of HCT‐116^wt^ and HCT‐116^p53−/−^ knockout mutant colon carcinomas, 518A2 melanoma, HeLa and mdr KB‐V1^Vbl^ cervical carcinomas, and human adult dermal fibroblast cells HDFa.

	IC_50_ [μm]^[a]^					
	HCT‐116^wt^	HCT‐116 p^53−/−^	518A2	HeLa	KB‐V1^Vbl^	HDFa
**4 a**	6.6±0.8	2.2±0.4	19.8±2.0	12.4±0.5	>50	24.6±3.4
**4 b**	16.4±0.2	8.4±0.3	7.9±0.8	23.7±1.1	5.9±1.3	9.0±1.3
**5 a**	1.1±0.3	0.6±0.1	5.0±0.3	3.6±0.7	0.6±0.2	5.8±0.9
**5 b**	1.3±0.6	0.4±0.1	2.9±0.5	1.8±0.4	2.2±0.2	5.9±0.2
**6 a**	0.2±0.02	0.05±0.001	0.4±0.1	0.3±0.02	4.6±0.2	1.4±0.2
**6 b**	0.3±0.03	0.2±0.05	5.5±0.4	3.6±0.4	0.7±0.2	3.2±0.4
auranofin	11.9±0.4	5.0±0.2	1.8±0.03	2.6±0.4	n.d.^[b]^	13.7±1.0

[a] Values are the means±standard deviation (SD) determined in four independent experiments and derived from dose–response curves after 72 h incubation by using the 3‐(4,5‐dimethylthiazol‐2‐yl)‐2,5‐diphenyltetrazolium bromide (MTT) assay. [b] Not determined.

### Intracellular localisation

**Figure 1:**
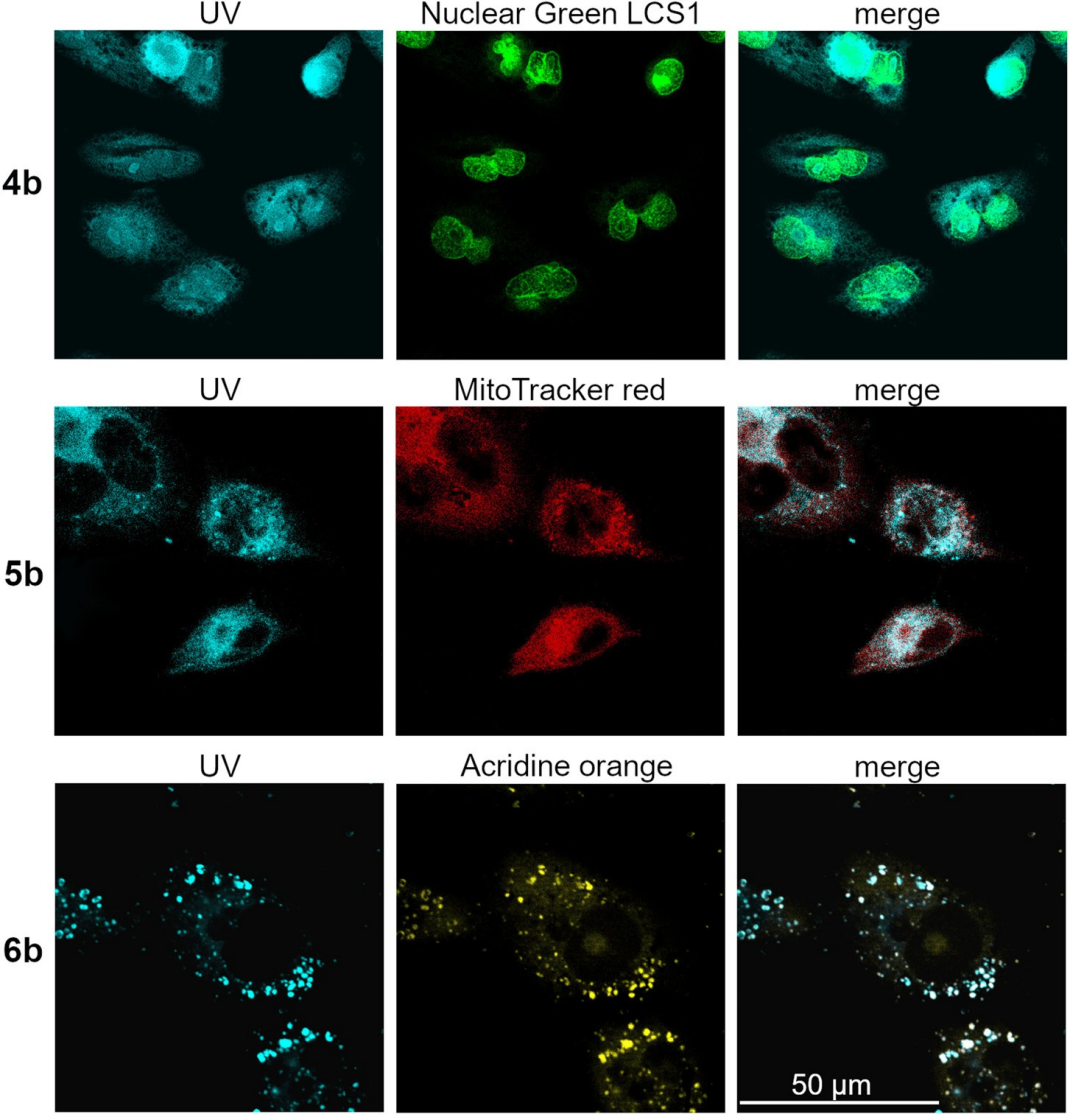
Confocal fluorescence microscopy images of 518A2 melanoma cells incubated for 30 min with 30 μm of complexes **4 b**–**6 b** (*λ*_ex_=350 nm and *λ*_em_=420–480 nm). The nuclei were counterstained with Nuclear Green LCS1 (abcam; *λ*_ex_=514 nm and *λ*_em_=520–535 nm), the mitochondria with MitoTracker^TM^ (Thermo Fisher; *λ*_ex_=580 nm and *λ*_em_=595–610 nm) and the lysosomes with acridine orange solution (5 μg mL^−1^, ABCR GmbH; *λ*_ex_=350 nm and *λ*_em_=600–660 nm). Images are representative of at least four independent experiments; 2000‐fold magnification.

The fluorescent complexes **4 b**, **5 b** and **6 b** were synthesised as easy‐to‐track analogues of complexes **4 a**, **5 a** and **6 a**, respectively. Well‐observable, flat 518A2 melanoma cells were treated with the **b** complexes, then counterstained with dyes specifically accumulating in particular cancer‐relevant cellular organelles, and eventually fixed and examined through confocal microscopy (Figure 1). By counterstaining with Nuclear Green, neutral chloride complex **4 b** could be localised in the area of the nucleus and to a minor degree in the cytoplasm. This is in line with reports on the nuclear accumulation of neutral gold(I) complexes bearing an aryl‐substituted NHC ligand.[^19^, ^20^] Many established first‐line anticancer drugs target cancer cell nuclei,^[21]^ yet suffer from therapeutic shortcomings, including off‐target side effects and an early onset of resistance, owing to insufficient nuclear accumulation.^[22]^ Against this background, the enrichment of new (NHC)Au^I^Cl complex **4 b** predominantly in cancer cell nuclei is remarkable. Cationic phosphane complex **5 b** accumulated in the mitochondria, as demonstrated by counterstaining of treated 518A2 cells with red mitochondria‐selective MitoTracker (Figure 1). Apparently, the DLC character of this complex favours accumulation in the negatively charged mitochondrial compartments over any potential DNA intercalation of the planar anthracene residue. Mitochondria are considered to be promising targets for cancer therapy. A distinct disruption of the MMP typically results in the induction of apoptosis. One of the pro‐apoptotic stimuli is an increased mitochondrial ROS production, which, in turn, causes disruption of the MMP.^[23]^ Cationic bis‐NHC complex **6 b** accumulated mainly in lysosomes within the cytoplasm. It should be noted that Gust et al. found an accumulation of all three [1,3‐diethyl‐4,5‐di(*p*‐fluorophenyl)imidazol‐2‐ylidene](L)gold(I) analogues of complexes **4 a**, **5 a** and **6 a** in the nuclei of MCF‐7 and HT‐29 cells upon a 24 h long exposure.^[24]^ So, the organelle‐selective accumulation of our **a** complexes after only 30 min might be a kinetic effect. The bottom row of Figure 1 shows confocal fluorescence microscopy images of 518A2 melanoma cells treated with complex **6 b** and lysotropic acridine orange, as well as the good match of the blue fluorescence of **6 b** (UV) with the orange fluorescence of the counterstained lysosomes. Lysosomes are the recycling centres of the cell and are involved in cellular digestion processes, such as autophagy, endocytosis and phagocytosis. Moreover, the release of lysosomal hydrolases, so called cathepsins, is involved in the induction of cell death.[^25^, ^26^] Cathepsins mediate caspase‐ and mitochondrion‐independent cell death, especially in cancer cells with mutations in genes involved in the classic apoptotic pathway, for example, the TP53 tumour suppressor gene.^[27]^

### Induction of cancer cell apoptosis

**Figure 2:**
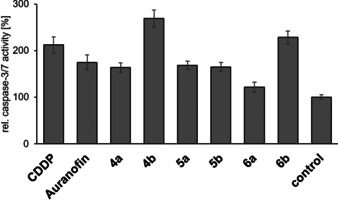
Induction of effector caspase‐3/‐7 activity in 518A2 melanoma cells after treatment with 5 μm **4**–**6** for 6 h, measured by means of the Apo‐ONE^®^ Homogenous Caspase‐3/7 Assay Kit (Promega). CDDP was used as a positive control. The vitality of cells was simultaneously tested by MTT assays and was >80 % for all experiments, except for complex **6 a** (70 %). All experiments were performed in triplicate and results quoted as means±SD. The solvent‐treated negative control was set to 100 %.

The majority of p53 mutations are missense mutations, as in the case of 518A2 melanoma cells,^[28]^ leading to the expression of dysfunctional p53 proteins with oncogenic activities intensifying malignant properties of cancer cells, such as clinical drug resistance.^[29]^ Because the p53‐independent induction of cancer cell apoptosis had been reported for auranofin[^17^, ^30^] and for (1,3‐diethylbenzimidazol‐2‐ylidene)gold(I) complexes,^[18]^ we investigated if complexes **4**–**6** also lead to an activation of apoptosis (Figure 2). Upon treatment of 518A2 melanoma cells with these complexes, the activation of effector caspases‐3 and ‐7 was observed, which we assumed to be p53 independent, given the results from our cytotoxicity studies. The treated cells showed the typical morphological signs of apoptosis, as well as translocalisation of phosphatidylserines to the outer leaflet of the plasma membrane, which indicated early rather than late apoptosis or necrosis (see the Supporting Information). Because about 50 % of all human tumours bear p53 mutations, drugs that induce p53‐independent programmed cell death are of particular interest.[^31^, ^32^]

### Mechanism of action of complexes 4 a and 4 b in the nucleus

**Figure 3:**
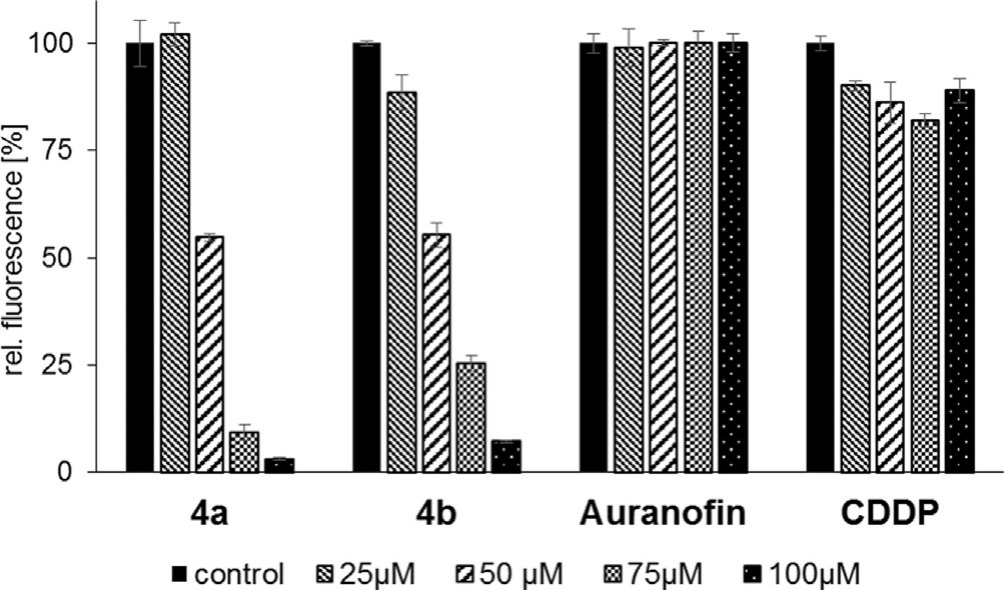
EtdBr saturation assays with 25, 50, 75 and 100 μm **4 a**, **4 b** and auranofin. CDDP was used as a positive control. Negative controls were treated with an equivalent amount of solvent (DMF or H_2_O). All experiments were carried out in triplicate with negative controls set to 100 %.

**Figure 4:**
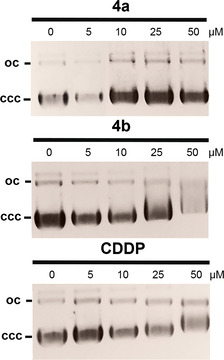
EMSAs with circular pBR322 plasmid DNA after 24 h treatment with complexes **4 a** or **4 b**, as visualised by UV radiation. CDDP was used as a positive control. Images are representative of at least two independent experiments.

The antiproliferative effect of CDDP and other platinum complexes is based mainly on their interaction with cellular DNA.[^3b^, ^33^] Because of the localisation of neutral complex **4 b** in the nuclear area, a potential DNA interaction of **4 b** and its close structural analogue **4 a** was examined by ethidium bromide (EtdBr) saturation assays (Figure 3) and electrophoretic mobility shift assays (EMSAs; Figure 4).

Addition of complexes **4 a** or **4 b** to linear, double‐stranded salmon sperm DNA led to a distinct concentration‐dependent displacement, and thus, to a reduction of the fluorescence of intercalated EtdBr, exceeding that caused by CDDP by far. This suggests a strong interaction of both complexes **4** with this DNA form, possibly associated with an alteration of the DNA morphology. Auranofin showed no such effect (Figure 3). In the EMSA with circular plasmid DNA, a slight relaxation, that is, despiralisation, of the covalently closed circular (ccc) DNA form for the benefit of the open circular (oc) form was observed after incubation with complex **4 a**, and a stronger relaxation after treatment with complex **4 b** (Figure 4). In contrast to CDDP, gold NHC complexes are known to bind non‐covalently to DNA, which may be the reason for their weaker effects in the EMSA.^[34]^

Although auranofin had previously been reported to interact neither with linear DNA nor with circular plasmid DNA,^[35]^ various other gold(I) complexes with readily displaceable ligands (e.g., Cl^−^) had shown affinity to different types of DNA.[^35^, ^36^] Irreparable DNA damage induces apoptosis, normally triggered by the tumour suppressor protein p53. However, apoptosis as a consequence of DNA damage caused by metal complexes had also been reported to proceed independently of p53,[^37^, ^38^] through the mitogen‐activated protein kinase (MAPK) signalling pathway involving JNK, p38 and ERK1/2.[^18^, ^37^] Whether complexes **4**, which we have found to induce cancer cell apoptosis and to be cytotoxic independently of functional p53, operate by a similar mechanism remains to be shown. At present, we cannot exclude that their reactions with further biologically relevant macromolecules might also play a role.^[39]^

### Mechanism of action of complexes 5 a and 5 b in mitochondria

Because cationic triphenylphosphane complex **5 b** was localised in the mitochondria of 518A2 melanoma cells, we anticipated a mitochondria‐associated mode of action for **5 b** and closely related complex **5 a**. The anticancer effect of auranofin, and several other gold(I) complexes, mainly relies on the inhibition of TrxR.[^40^, ^41^] TrxRs, which catalyse the reduced nicotinamide adenine dinucleotide phosphate (NADPH)‐dependent reduction of the redox protein thioredoxin (Trx) and other compounds, are key enzymes for cellular protection against oxidative stress.^[42]^ To date, three different isoforms of TrxR are known: cytosolic TrxR1, mitochondrial TrxR2 and testis‐specific TrxR3.^[43]^ Gold complexes, such as auranofin, are thought to inhibit TrxRs by releasing monovalent Au^I^ species, which bind to selenocysteine residues in the active site of the enzyme.^[44]^ This is in line with reports that mono‐NHC gold(I) complexes with good leaving groups, such as halides or phosphanes, are better TrxR inhibitors than bis‐NHC complexes.^[45]^ For instance, sub‐micromolar IC_50_ values were reported by Gust et al. for donor‐substituted (1,3‐diethyl‐4,5‐diarylimidazol‐2‐ylidene)(PPh_3_)gold(I) complexes,^[24]^ and by Ott et al. for benzimidazol‐2‐ylidene analogues,[^15^, ^46^] whereas few inhibitory bis(1,3‐diarylimidazol‐2‐ylidene) complexes have been reported, to date.[^45^, ^47^]

**Figure 5:**
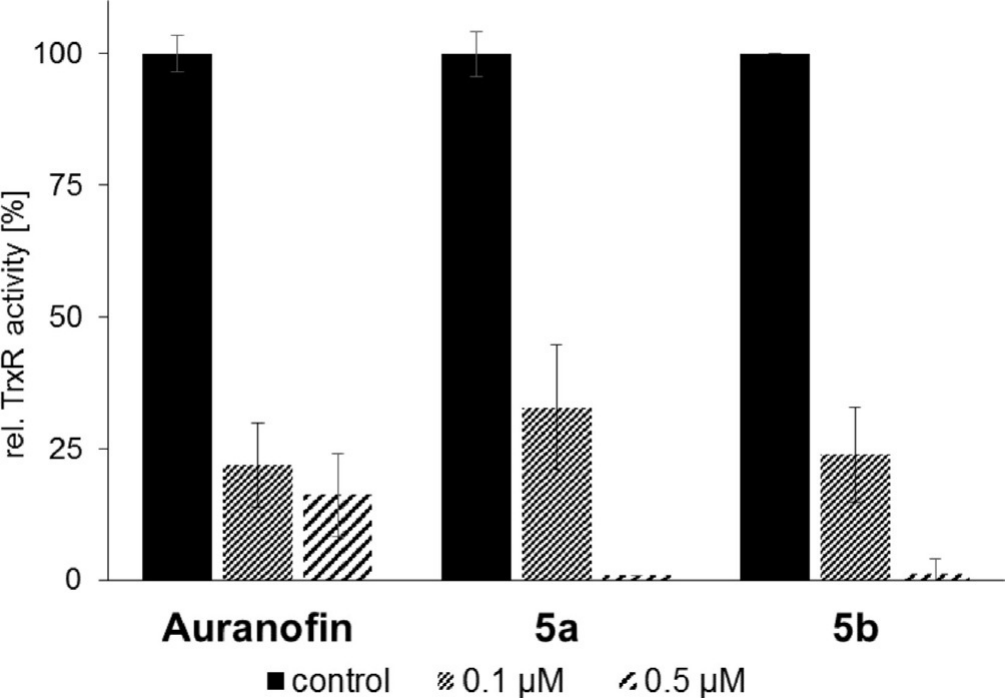
Concentration‐dependent inhibition of TrxR activity in cell lysates of 518A2 melanoma cells by gold(I) complexes **5 a** and **5 b**, and auranofin as a positive control. TrxR‐independent substrate reduction was accounted for by experiments in the presence and absence of the specific TrxR inhibitor aurothiomalate. All values are means±SD of at least three independent experiments with negative controls set to 100 %.

If applied in low sub‐micromolar concentrations, complexes **5 a** and **5 b** strongly inhibited the panTrxR activity in colorimetric TrxR microplate assays with 5,5′‐dithiobis(2‐dinitrobenzoic acid (DTNB; Ellman's reagent) as a substrate (Figure 5). Because many tumours have elevated TrxR levels,^[43]^ and tumour cells are more sensitive to oxidative stress, due to their a priori high intracellular ROS levels relative to non‐malignant cells, TrxR are interesting targets for selective antitumour therapy.

**Figure 6:**
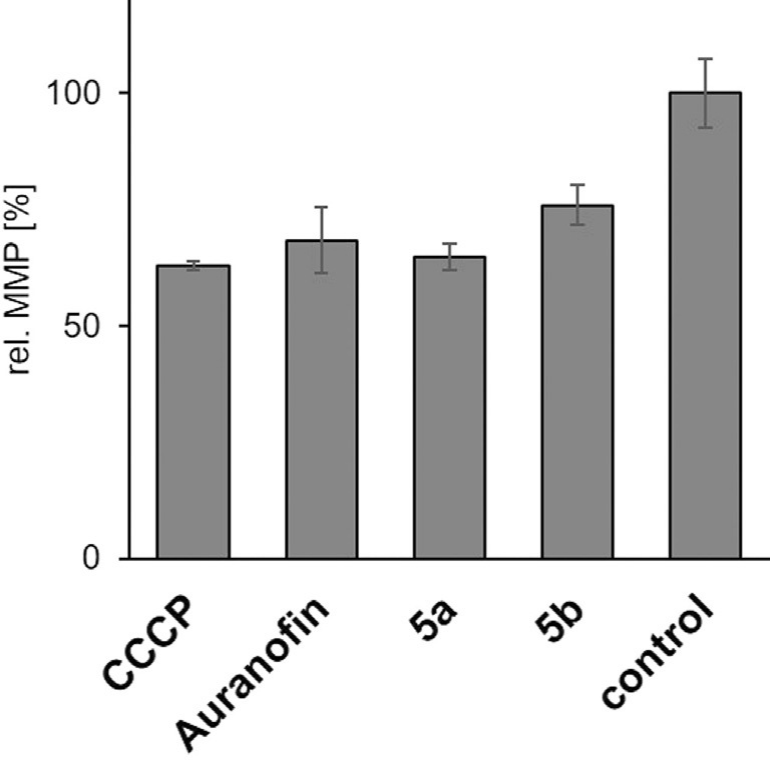
Relative MMP in 518A2 melanoma cells after treatment (45 min) with complexes **5 a** and **5 b** (10 μm each). Carbonylcyanide‐*m*‐chlorophenylhydrazone (CCCP) and auranofin (10 μm, each) were used as positive controls and solvent‐treated negative controls were set to 100 %. Assays were carried out in triplicate.

TrxR inhibition generally leads to an accumulation of oxidised Trx and ROS in mitochondria, resulting in an increase of mitochondrial permeability.^[40]^ Upon treatment of 518A2 melanoma cells with complexes **5 a** and **5 b**, we observed a distinct reduction of the MMP through a fluorescence‐based microplate assay (Figure 6), exceeding that induced by auranofin, which is in keeping with their stronger TrxR inhibition.

**Figure 7:**
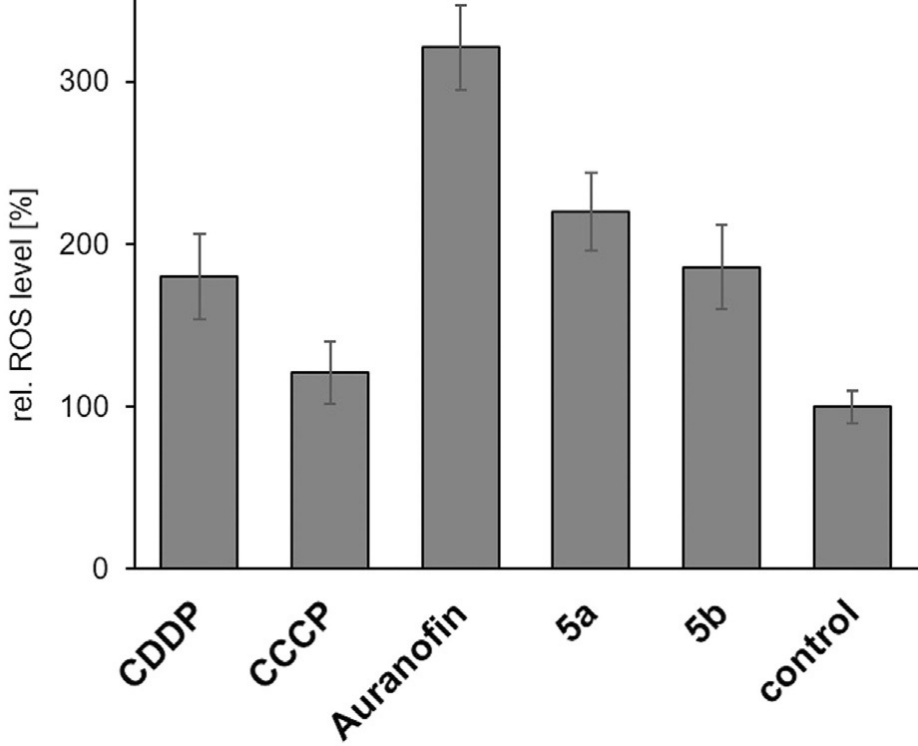
Influence of gold(I) complexes **5 a** and **5 b**, and auranofin (10 μm each), as well as CCCP (10 μm) as a positive control, on the levels of ROS in 518A2 melanoma cells, as determined by fluorescence‐based DCFH‐DA assays after an incubation time of 1 h. Negative controls were treated identically with solvent. All values are mean values±SD from at least four independent experiments with negative controls set to 100 %.

We confirmed these results by an assessment of the intracellular ROS concentrations after treatment of 518A2 melanoma cells with auranofin, CCCP and complexes **5 a** and **5 b** using the cell permeant, fluorogenic dye 2′,7′‐dichlorofluorescein diacetate (DCFH‐DA). After diffusion into the cells, DCFH‐DA is deacetylated by cellular esterases to a non‐fluorescent compound, which is later oxidised by hydroxyl, peroxyl or other ROS to the intensely fluorescent 2′,7′‐dichlorofluorescein (DCF), detectable by fluorescence spectroscopy (Figure 7).

We conclude that the cytotoxicity of complexes **5** originates mainly from their inhibition of TrxR in the mitochondria of cancer cells and the subsequent alteration of the intracellular ROS equilibrium.^[40]^ Elevated concentrations of hydrogen peroxide and oxidised Trx2 affect further intra‐mitochondrial targets, leading to the opening of the mitochondrial permeability transition pore and/or to an increase of the permeability of the outer membrane.[^9^, ^48^] As a result, hydrogen peroxide is released into the cytosol where it oxidises cytosolic Trx1 irreversibly, due to the inhibition of TrxRs. The elevated levels of hydrogen peroxide and oxidised Trx in the cytosol then activate various signalling pathways, eventually leading to apoptosis, which is likely to be dependent on p38/ERK1/2, rather than p53, as shown for auranofin.[^40^, ^49^] Because cancer cells, unlike non‐malignant cells, are not normally susceptible to mitochondrial membrane permeability transition, the induction of this condition by mitochondria‐targeting complexes, such as **5**, could be exploited in a therapeutic context.^[50]^

### Mechanism of action of complexes 6 a and 6 b in lysosomes

**Figure 8:**
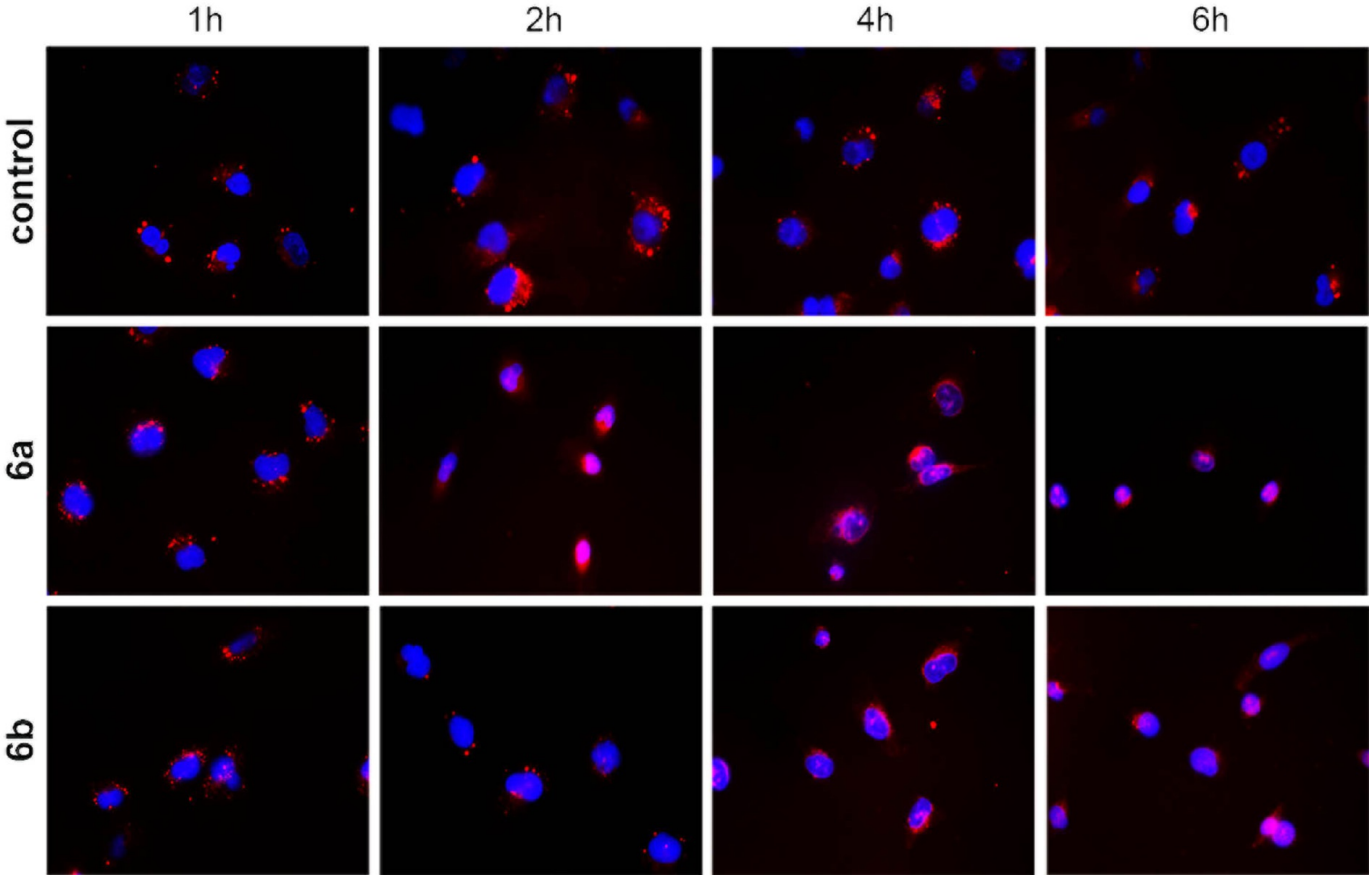
Fluorescence microscopy images of 518A2 melanoma cells treated with solvent (DMF), or complexes **6 a** (0.4 μm) or **6 b** (5.5 μm), for 1, 2, 4 or 6 h under standard cell‐culture conditions; 30 min before each time interval ended, cells were stained with Lysosomal Staining Reagent Orange (Abcam). Nuclear counterstaining was performed by using blue 4′,6‐diamidino‐2‐phenylindole (DAPI). Images are representative of at least ten independent measurements at 400‐fold magnification.

The cationic bis‐NHC complex **6 b** was localised in the lysosomes of 518A2 melanoma cells. Lysosomes mediate the degradation of macromolecules of intracellular origin or those that are internalised by endocytosis or phagocytosis.^[51]^ These single‐membrane acidic organelles (pH 4.5–4.8) are involved in various cellular pathways and different types of cell death, and their functionality is thus inevitable for cellular homeostasis.^[51]^ Various forms of cellular stress lead to lysosomal swelling and lysosomal membrane permeabilisation (LMP), resulting in the release of intralysosomal cargo into the cytoplasm.^[51]^ Amongst others, cathepsins B and D are released into the cytoplasm under stress, where they induce different forms of cell death, including the p53‐independent, lysosome‐dependent apoptotic cell death.[^26^, ^31^, ^51^, ^52^] To detect a potential induction of LMP by complexes **6**, we performed a time‐dependent staining of lysosomes in solvent‐ and complex‐treated 518A2 melanoma cells (Figure 8). Because the cytotoxicity of both complexes against 518A2 cells in MTT assays was quite different (IC_50_(**6 a**)=0.4 μm, IC_50_(**6 b**)=5.5 μm), we adjusted their concentrations accordingly to ensure a sufficient cell viability. The incubation with either complex **6 a** or **6 b** led to an induction of LMP. The lysotropic orange dye used in this assay selectively accumulates in intact acidic lysosomes. If LMP occurs, the dye is released into the cytosol and the fluorescence of defined lysosomal compartments disappears. As expected, complex **6 a**, which had proved to be more active in MTT assays, also led to faster lysosomal disruption after only 2 h of incubation. Cells treated with **6 b** showed first signs of LMP only after 4 h of treatment.

## Conclusion

The [4‐(anthracen‐9‐yl)‐1,3‐diethyl‐5‐phenylimidazol‐2‐ylidene] (L)gold(I) complexes **4 b**, **5 b**, and **6 b** accumulated quickly in different compartments of 518A2 melanoma cells, that is, neutral chlorido complex **4 b** in the nuclei, cationic phosphane complex **5 b** in mitochondria and large delocalised cationic bis‐NHC complex **6 b** in the lysosomes. The analogous **a** series of complexes carried a slightly different 4,5‐diarylimidazol‐2‐ylidene ligand. The fact that all couples **4 a**/**4 b**, **5 a**/**5 b** and **6 a**/**6 b** afforded similar results in cytotoxicity tests with cancer cells, and in tests on targets typically found at the identified sites of accumulation, supports the assumption that **a** complexes localise similarly to the **b** complexes, and that the nature of ligand L, which is responsible for the charge, size and lipophilicity of the complex, is decisive for the site of accumulation. However, this phenomenon might be limited to divalent gold(I)–NHC or even to (imidazol‐2‐ylidene)gold(I) complexes because a comparable series of *cis*‐[bis(1,3‐dibenzylimidazol‐2‐ylidene)]Cl(L)Pt^II[14]^ and (1,3‐diethylbenzimidazol‐2‐ylidene)(L)gold(I) complexes,^[15]^ carrying the same ligands L (Cl, PPh_3_ or the same NHC ligand), were previously shown to accumulate in mitochondria, regardless of the charge of the complex and the nature of ligand L. The different distributions of DLC complexes **5** (in mitochondria) and **6** (in lysosomes) is explicable by the higher molecular weight and steric demand of the latter, which are too large for embedding in the mitochondrial membrane, and thus, are dealt with by the cellular “waste‐to‐energy plants”, the lysosomes. Once fully understood, the concept of controlling the intracellular distribution of metallodrugs by the choice of secondary ligands and charge of the complex could be exploited in rational drug design.

For the mode of action of new complexes **4**–**6**, we found an eventual induction of p53‐independent apoptotic cell death, which was initiated by different effects of the three complex types at their respective sites of accumulation.

## Conflict of interest

The authors declare no conflict of interest.

## Supporting information

As a service to our authors and readers, this journal provides supporting information supplied by the authors. Such materials are peer reviewed and may be re‐organized for online delivery, but are not copy‐edited or typeset. Technical support issues arising from supporting information (other than missing files) should be addressed to the authors.

Supplementary
