# Supplementary material for: Guided Antitumoural Drugs: (Imidazol‐2‐ylidene)(L)gold(I) Complexes Seeking Cellular Targets Controlled by the Nature of Ligand L
Source: Chemistry. 2021 Feb 8;27(15):5003–10. doi: 10.1002/chem.202005451 (PMC7986617; doi:10.1002/chem.202005451)
Supplement: Supplementary file 1 — Supplementary [file CHEM-27-5003-s001.pdf]

# Chemistry–A European Journal

Supporting Information


## Guided Antitumoural Drugs: (Imidazol-2-ylidene)(L)gold(I) Complexes Seeking Cellular Targets Controlled by the Nature of Ligand L


Sofia I. Bär[+], Madeleine Gold[+], Sebastian W. Schleser, Tobias Rehm, Alexander Bär, Leonhard Köhler, Lucas R. Carnell, Bernhard Biersack, and Rainer Schobert*[a]

## Author Contributions

S.B. Formal analysis: Lead; Investigation: Lead; Methodology: Lead; Project administration: Lead; Writing – original draft: Lead
M.G. Formal analysis: Equal; Investigation: Equal; Methodology: Equal; Project administration: Equal; Writing – original draft: Equal
S.S. Formal analysis: Supporting; Investigation: Supporting; Methodology: Supporting
T.R. Formal analysis: Supporting; Investigation: Supporting; Methodology: Supporting
A.B. Formal analysis: Supporting; Investigation: Supporting; Methodology: Supporting
L.K. Formal analysis: Supporting; Investigation: Supporting; Methodology: Supporting
L.C. Formal analysis: Supporting; Methodology: Supporting
B.B. Formal analysis: Supporting; Investigation: Supporting; Methodology: Supporting; Project administration: Supporting.

## Table of Contents

**Experimental Procedures** .......... 3

*Chemical Synthesis and Analytics* .......... 3

General. .......... 3

5-(Anthracen-9-yl)-1-ethyl-4-phenylimidazole (2). .......... 3

4-(Anthracen-9-yl)-1,3-diethyl-5-phenylimidazolium tetrafluoroborate (3b). .......... 3

Chlorido-[4-(anthracen-9-yl)-1,3-diethyl-5-phenylimidazol-2-ylidene]gold(I) (4b). .......... 3

[1,3-Diethyl-5-(4-methoxyphenyl)-4-(3,4,5-trimethoxyphenyl)imidazol-2-ylidene](triphenylphosphane)gold(I) tetrafluoroborate (5a). .......... 3

[4-(Anthracen-9-yl)-1,3-diethyl-5-phenylimidazol-2-ylidene](triphenylphosphane)gold(I) tetrafluoroborate (5b). .......... 4

Bis[4-(anthracen-9-yl)-1,3-diethyl-5-phenylimidazol-2-ylidene]gold(I) tetrafluoroborate (6b). .......... 4

NMR Spectra .......... 4

*Biochemical Evaluation* .......... 9

Cell lines and culture conditions. .......... 9

Intracellular localisation of gold complexes. .......... 10

Caspase-3/7 activation assay. .......... 10

Detection of morphological signs of apoptosis. .......... 10

Annexin-V-FITC/PI staining. .......... 10

Ethidium bromide saturation assay. .......... 10

Electrophoretic mobility shift assay (EMSA). .......... 10

Inhibition of thioredoxin reductase (TrxR) activity. .......... 11

Mitochondrial membrane potential. .......... 11

Determination of intracellular concentration of reactive oxygen species (DCFH-DA assay). .......... 11

Lysosomal integrity .......... 11

Stability testing via NMR spectroscopy. .......... 11

Tubulin polymerisation assay. .......... 11

Cell cycle analysis. .......... 12

**Results** .......... 13

Influence on cellular morphology .......... 13

Apoptosis detection using Annexin V-FITC and PI .......... 14

Stability testing via NMR spectroscopy .......... 15

Interaction with tubulin .......... 18

Influence on the cell cycle of 518A2 melanoma cells .......... 18

**References** .......... 19

**Author Contributions** .......... 19

## Experimental Procedures

*Chemical Synthesis and Analytics*

**General.**

Melting points (uncorrected): GALLENKAMP; IR spectra: PERKIN-ELMER Spectrum One FT-IR spectrophotometer with ATR sampling unit; Nuclear magnetic resonance (NMR) spectra: BRUKER DRX 500 Hz spectrometer, chemical shifts are given in parts per million (δ) downfield from tetramethylsilane as internal standard for $^{1}$H and $^{13}$C; Mass spectra: VARIAN MAT 311A (EI), WATERS UPLC-Q-TOF (ESI), ThermoFisher UPLC/Orbitrap MS system (HRMS-ESI); All starting compounds were purchased from ALDRICH and used without further purification. The known compounds **3a**[1], **4a**[1] and **6a**[1] were prepared according to literature procedures.

**5-(Anthracen-9-yl)-1-ethyl-4-phenylimidazole (2)**. A solution of 9-formylanthracene (700 mg, 3.39 mmol) in ethanol (50 mL) was treated with 2M $EtNH_2$/THF (8.49 mL, 17.0 mmol). Acetic acid (970 μL) was added and the reaction mixture was refluxed for 2 h. After cooling to room temperature, phenyl-TosMIC **1** (1.39 g, 5.09 mmol) and $K_2CO_3$ (1.88 g, 13.6 mmol) were added and refluxed again for 6 h. The solvent was evaporated, the residue was dissolved in ethyl acetate (100 mL) and washed with water (100 mL) and brine (100 mL), dried over $Na_2SO_4$, filtered and the filtrate was concentrated in vacuum. The residue was purified by column chromatography (silica gel 60; EtOAc/MeOH: 97/3). Yield: 726 mg (2.08 mmol, 61%); yellow solid; $R_f$ = 0.64; $\nu_{max}$/cm$^{-1}$: 3053, 2977, 2931, 1623, 1599, 1517, 1501, 1458, 1442, 1398, 1373, 1352, 1343, 1314, 1244, 1224, 1175, 1163, 1135, 1117, 1056, 1067, 1011, 983, 951, 917, 899, 852, 813, 799, 771, 744, 715, 728, 692; $^{1}$H NMR (500 MHz, $CDCl_3$): δ 1.01 (3 H, t, J = 7.3 Hz), 3.45 (2 H, q, J = 7.3 Hz), 6.96-6.98 (3 H, m), 7.29-7.31 (2 H, m), 7.38-7.41 (2 H, m), 7.48-7.51 (2 H, m) 7.64 (2 H, d, J = 8.7 Hz), 7.91 (1 H, s), 8.10 (2 H, d, J = 8.7 Hz), 8.64 (1 H, s); $^{13}$C NMR (126 MHz, $CDCl_3$): δ 16.4, 40.1, 123.9, 124.5, 125.4, 125.6, 125.7, 126.1, 126.9, 128.1, 128.8, 128.9, 131.5, 134.0, 134.5, 136.8, 140.0.

**4-(Anthracen-9-yl)-1,3-diethyl-5-phenylimidazolium tetrafluoroborate (3b)**.

Compound **2** (500 mg, 1.43 mmol) was dissolved in acetonitrile (100 mL) and iodoethane (6.34 mL) was added. The reaction mixture was stirred at 85 °C for 48 h. The solvent was evaporated and the remainder was crystallised from $CH_2Cl_2$/*n*-hexane at 4 °C. Yield: 689 mg (1.37 mmol, 95%); yellow solid; $v_{max}$/cm$^{-1}$: 3420, 3118, 3027, 2979, 1622, 1592, 1556, 1520, 1499, 1443, 1386, 1348, 1263, 1193, 1159, 1091, 1074, 1023, 1012, 962, 932, 897, 854, 794, 774, 740, 700; $^{1}$H NMR (500 MHz, $CDCl_3$): $^{1}$H NMR (500 MHz, $CDCl_3$): δ 1.28 (3 H, t, J = 7.3 Hz), 1.68 (3 H, t, J = 7.3 Hz), 3.91 (2 H, q, J = 7.3 Hz), 4.52 (2 H, q, J = 7.3 Hz), 7.13-7.23 (5 H, m), 7.50-7.55 (2 H, m), 7.59 (2 H, t, J = 8.1 Hz), 7.65 (2 H, d, J = 8.7 Hz) 8.06 (2 H, d, J = 8.4 Hz), 8.62 (1 H, s), 10.83 (1 H, s);$^{13}$C NMR (126 MHz, $CDCl_3$): δ 15.8, 15.9, 43.6, 44.1, 117.0, 124.4, 124.9, 126.0, 128.3, 128.4, 129.1, 129.2, 129.4, 130.4, 131.0, 131.2, 132.0, 133.9. The resulting 4-(anthracen-9-yl)-1,3-diethyl-5-phenylimidazolium iodide (41 mg, 0.081 mmol) was dissolved in acetone (10 mL) and $NaBF_4$ (13 mg, 0.122 mmol) was added. The reaction mixture was stirred at room temperature for 24 h. After filtration through $Mg_2SO_4$ the filtrate was concentrated in vacuum and dried. Yield: 38 mg (0.081 mmol, 100%); yellow solid; $v_{max}$/cm$^{-1}$: 3464, 3039, 2982, 1622, 1557, 1520, 1499, 1444, 1387, 1349, 1333, 1305, 1263, 1193, 1065, 1023, 963, 932, 897, 854, 774, 740, 727, 700; $^{1}$H NMR (300 MHz, $CDCl_3$): δ 1.24 (3 H, t, J = 7.3 Hz), 1.65 (3 H, t, J = 7.3 Hz), 3.88 (2 H, q, J = 7.3 Hz), 4.49 (2 H, q, J = 7.3 Hz), 7.0-7.2 (5 H, m), 7.4-7.6 (4 H, m), 7.67 (2 H, d, J = 8.6 Hz), 8.01 (2 H, d, J = 8.4 Hz), 8.58 (1 H, s), 10.66 (1 H, s); $^{13}$C NMR (75.5 MHz, $CDCl_3$): δ 15.7, 15.8, 43.5, 44.0, 117.0, 124.3, 124.9, 125.8, 128.2, 128.3, 129.0, 129.1, 129.4, 130.2, 130.9, 131.0, 131.9, 133.9, 137.2; $^{11}$B NMR (96.3 MHz, $CDCl_3$): δ -0.76.

**Chlorido-[4-(anthracen-9-yl)-1,3-diethyl-5-phenylimidazol-2-ylidene]gold(I) (4b)**.

Compound **3b** (100 mg, 0.198 mmol) was dissolved in $CH_2Cl_2$ (5 mL) and treated with $Ag_2O$ (27.6 mg, 0.119 mmol). The mixture was stirred in darkness at room temperature for 5 h. Chloro(dimethylsulfide)gold(I) (64.2 mg, 0.218 mmol) and LiCl (84 mg, 1.98 mmol) were added and the reaction mixture was stirred for additional 24 h. The crude product was crystallised from $CH_2Cl_2$/*n*-hexane at 4 °C. Yield: 111 mg (0.182 mmol, 92%); yellowish solid of of mp > 250 °C (dec.); $v_{max}$ (ATR)/cm$^{-1}$: 3051, 2976, 2933, 1622, 1500, 1461, 1427, 1443, 1414, 1344, 1294, 1213, 1115, 1088, 1025, 1013, 997, 962, 933, 896, 851, 819, 775, 757, 727, 699, 652, 607, 582; $^{1}$H NMR(500 MHz, $CDCl_3$): δ 0.98 (3 H, t, J = 7.2 Hz), 1.45 (3 H, t, J = 7.1 Hz), 3.81 (2 H, q, J = 7.2 Hz), 4.37 (2 H, q, *J* = 7.2 Hz), 7.07-7.16 (5 H, m), 7.45-7.55 (4 H, m), 7.60 (2 H, d, J = 8.4 Hz), 8.00-8.05 (2 H, m), 8.55 (1 H, s); $^{13}$C NMR (125 MHz, $CDCl_3$): δ 17.0, 17.2, 44.7, 44.8, 120.4, 124.7, 125.7, 127.0, 127.5, 127.6, 128.7, 129.1, 129.2, 129.3, 129.4, 130.2, 131.1, 132.0, 133.0, 170.3; *m/z* (ESI, %) 614.2 [$M^+$+$CH_3CN$] (100). HRMS (ESI) m/z ((M-Cl+MeCN)$^+$) found 614.18508; calcd. 614.18650.

**[1,3-Diethyl-5-(4-methoxyphenyl)-4-(3,4,5-trimethoxyphenyl)imidazol-2-ylidene](triphenylphosphane)gold(I) tetrafluoroborate (5a)**.

Complex **4a** (112 mg, 0.178 mmol) was dissolved in acetone (10 mL) and $NaBF_4$ (36 mg, 0.33 mmol) and triphenylphosphane (61 mg, 0.23 mmol) were added. The reaction mixture was stirred at room temperature for 24 h. The suspension was filtered, the filtrate concentrated in vacuum, and the residue recrystallized from acetone/*n*-hexane. Yield: 132 mg (0.14 mmol, 79%); colorless solid of mp = 107-110 °C; Elemental analysis ($C_{41}H_{43}AuBF_4N_2O_4P$, %) found C 52.14 H 4.47 N 2.93; calcd. C 52.25, H 4.60, N 2.97. $v_{max}$ (ATR)/cm$^{-1}$: 3056, 2936, 2836, 1607, 1581, 1516, 1504, 1463, 1437, 1415, 1331, 1292, 1248, 1179, 1124, 1099, 1050, 1024, 997, 887, 839, 811, 748, 711, 693 $^{1}$H NMR (500 MHz, $CDCl_3$): δ 1.42 (3H, t, *J* = 7.3 Hz), 1.49 (3H, *J* = 7.2 Hz), 3.76 (3 H, s), 3.82 (3 H, s), 3.85 (3 H, s), 4.27 (2 H, q, *J* = 7.1 Hz), 4.35 (2 H, q, J = 7.1 Hz), 6.44 (3 H, s), 6.92 (6H, vd); 7.21 (3H, vd) 7.46 (15H, m) $^{13}$C NMR

(125 MHz, $CDCl_3$): δ 17.5, 17.8, 44.3, 44.6, 55.3, 56.3, 60.9, 107.7, 114.3, 128.8, 128.9, 130.8, 131.9, 133.9, 134.0; $^{31}P$ NMR (202 MHz, $CDCl_3$): δ 33.8; *m/z* (ESI, %) 855.0 (100) [M$^+$], 721.4 (22). HRMS (ESI) m/z (M$^+$) found 855.2577; calcd. 855.2620.

**[4-(Anthracen-9-yl)-1,3-diethyl-5-phenylimidazol-2-ylidene](triphenylphosphane)gold(I) tetrafluoroborate (5b)**.
Compound **3b** (100 mg, 0.198 mmol) in dry $CH_2Cl_2$ (5 mL) was treated with KO*t*Bu (27 mg, 0.238 mmol) and $(PPh_3)AuCl$ (98 mg, 0.198 mmol). The mixture was stirred at room temperature for 24. The crude product was filtered and crystallised from $CH_2Cl_2$/*n*-hexane at 4 °C. Yield: 166 mg (0.191 mmol, 96%); white solid of of mp = 165 °C; $\nu_{max}$ (ATR)/cm$^{-1}$: 3051, 2981, 1977, 1622, 1464, 1432, 1345, 1262, 1095, 1025, 895, 849, 775, 737, 690, 607, 565; $^1H$ NMR (500 MHz, $CDCl_3$): δ 1.14 (3 H, t, J = 7.2 Hz), 1.59 (3 H, t, J = 7.2 Hz), 3.92 (2 H, q, J = 7.2 Hz), 4.55 (2 H, q, *J* = 7.2 Hz), 7.10-7.16 (3 H, m), 7.19-7.21 (2 H, m), 7.35-7.39 (6 H, m), 7.42-7.45 (3 H, m), 7.48-7.52 (8 H, m), 7.56-7.59 (2 H, m), 7.73 (2 H, d, J = 8.4 Hz), 8.03 (2 H, d, J = 8.4 Hz), 8.57 (1H, s); $^{13}C$ NMR (125 MHz, $CDCl_3$): δ 17.6, 17.8, 44.8, 45.1, 120.2, 125.0, 125.7, 127.4, 127.7, 128.8, 128.9, 129.0, 129.4, 129.6, 130.2, 130.6, 131.1, 132.1, 132.2, 133.4 133.9, 134.0, 134.1, 183.9; $^{31}P$ NMR (202 MHz, $CDCl_3$): δ 30.3; *m/z* (ESI, %) 949.4 (100), 835.2 (7) [M$^+$], 721.2 (40). HRMS (ESI) m/z (M$^+$) found 835.2476; calcd. 835.2511.

**Bis[4-(anthracen-9-yl)-1,3-diethyl-5-phenylimidazol-2-ylidene]gold(I) tetrafluoroborate (6b)**.
Compound **3b** (100 mg, 0.198 mmol) was dissolved in $CH_2Cl_2$/methanol (1:1, 80 mL) and $Ag_2O$ (50.1 mg, 0.216 mmol) was added. The reaction mixture was stirred in the dark at room temperature for 5 h. Chloro(dimethylsulfide)gold(I) (33.9 mg, 0.115 mmol) was added and the reaction mixture was stirred for additional 24 h. The suspension was filtered, the filtrate was concentrated in vacuum and the residue was redissolved in $CH_2Cl_2$, filtered over $MgSO_4$/Celite, and the filtrate was concentrated in vacuum and the residue dried in vacuum. Yield: 79 mg (0.080 mmol, 88%); reddish solid of mp > 250 °C (dec.); $\nu_{max}$/cm$^{-1}$: 3052, 2965, 2924, 1623, 1595, 1520, 1498, 1460, 1443, 1407, 1378, 1345, 1294, 1260, 1218, 1161, 1088, 1050, 1012, 988, 961, 917, 896, 852, 774, 757, 737, 698; $^1H$ NMR (500 MHz, $CDCl_3$): δ 1.13 (6 H, t, J = 7.2 Hz), 1.59 (6 H, t, J = 7.2 Hz), 3.88 (4 H, q, J = 7.2 Hz), 4.49 (4 H, q, J = 7.2 Hz), 7.08-7.22 (10 H, m), 7.51 (4 H, t, J = 8.7 Hz), 7.58 (4 H, t, J = 8.7 Hz), 7.68 (4 H, d, J = 8.4 Hz), 8.04 (4 H, d, J = 8.4 Hz), 8.58 (2 H, s); $^{13}C$ NMR (126 MHz, $CDCl_3$): δ 17.6, 17.8, 44.7, 44.8, 119.8, 124.6, 124.7, 125.8, 127.1, 127.8, 127.9, 128.9, 129.1, 129.4, 129.5, 129.6, 130.4, 131.1, 131.2, 132.2, 134.0, 183.6; *m/z* (ESI, %) 949.4 [M$^+$] (100). HRMS (ESI) m/z (M$^+$) found 949.35035; calcd. 949.35390.

**NMR Spectra**

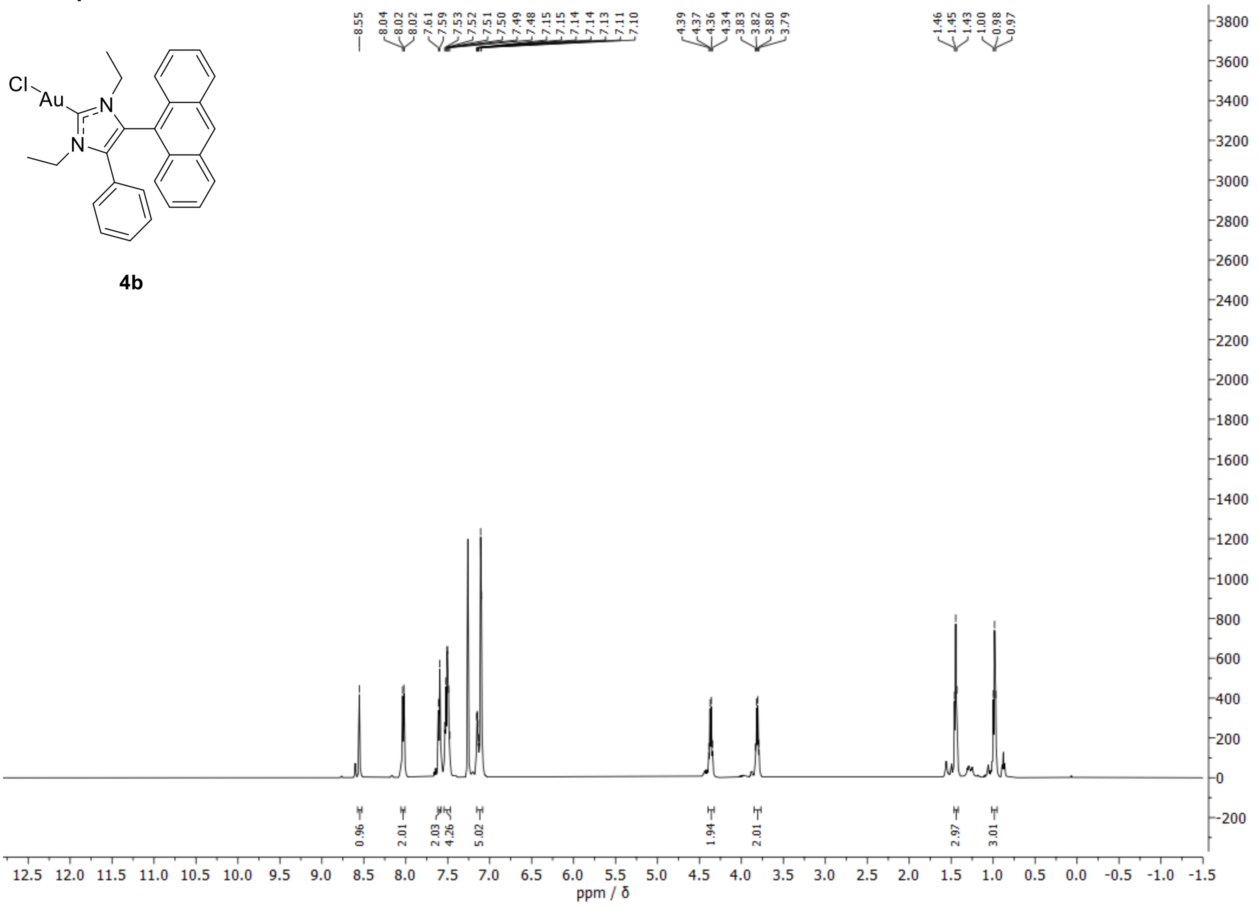


**Figure 1**: $^1H$ NMR (500 MHz, $CDCl_3$) spectrum of **4b.**

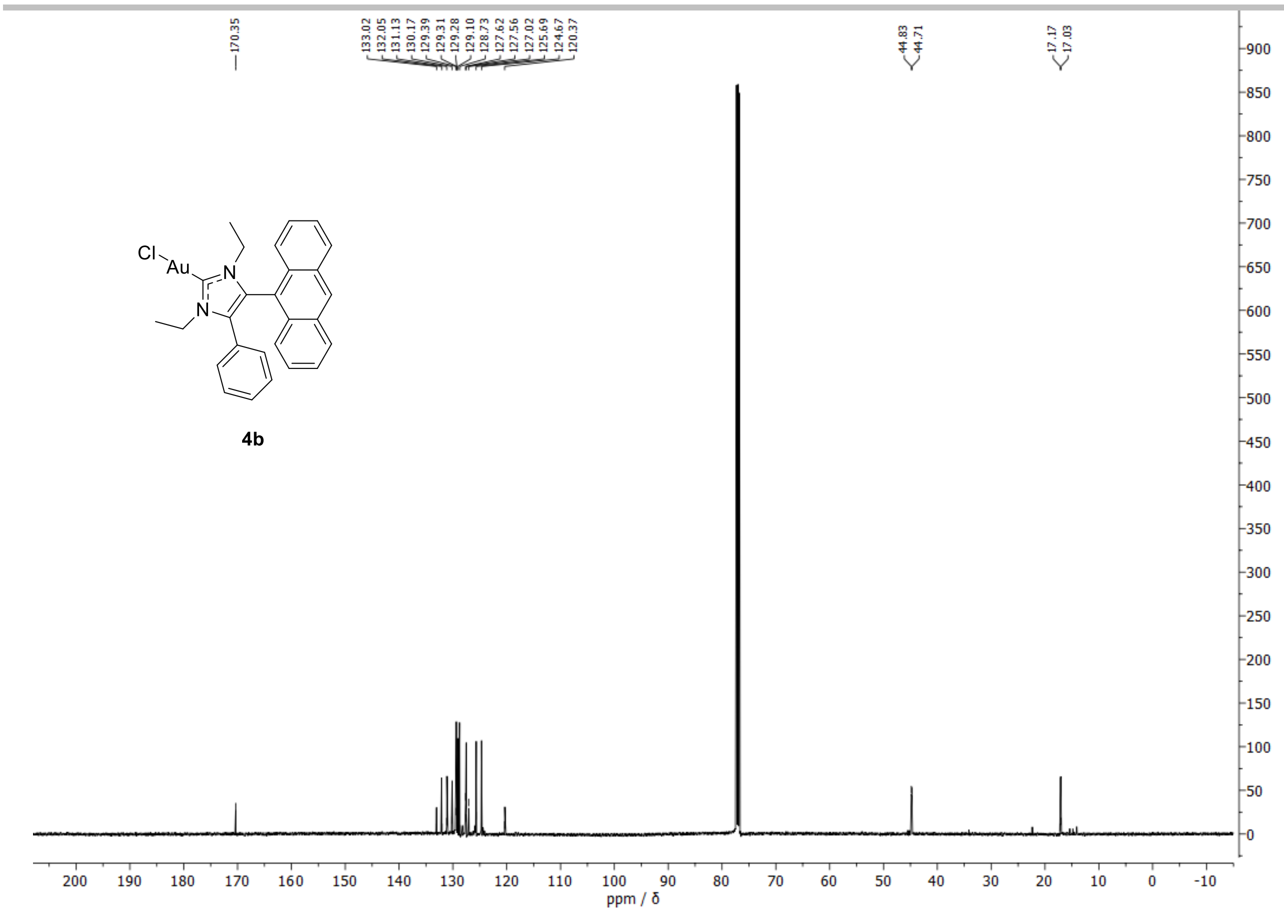


**Figure 2**: $^{13}C$ NMR (125 MHz, $CDCl_3$) spectrum of **4b.**

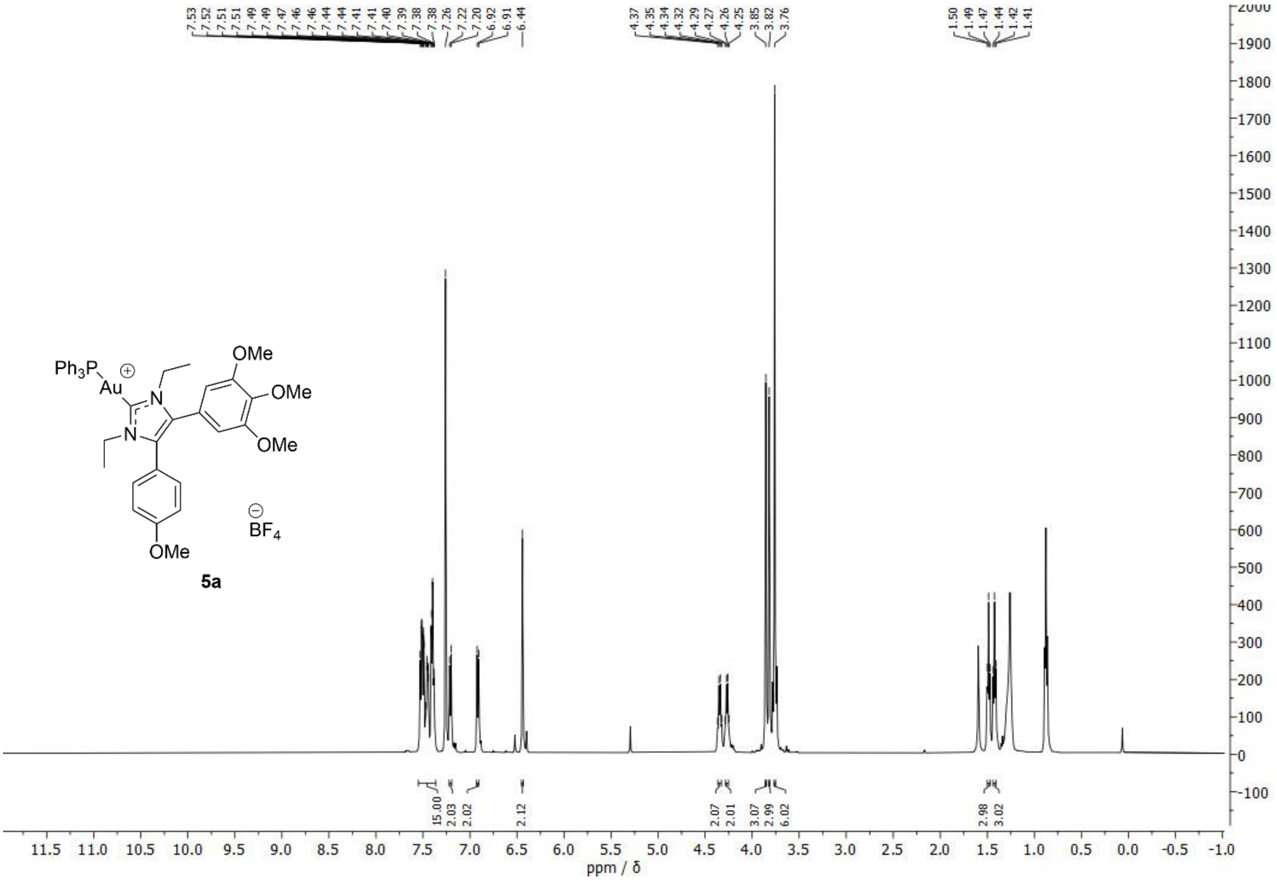


**Figure 3**: $^{1}H$ NMR (500 MHz, $CDCl_3$) spectrum of **5a.**

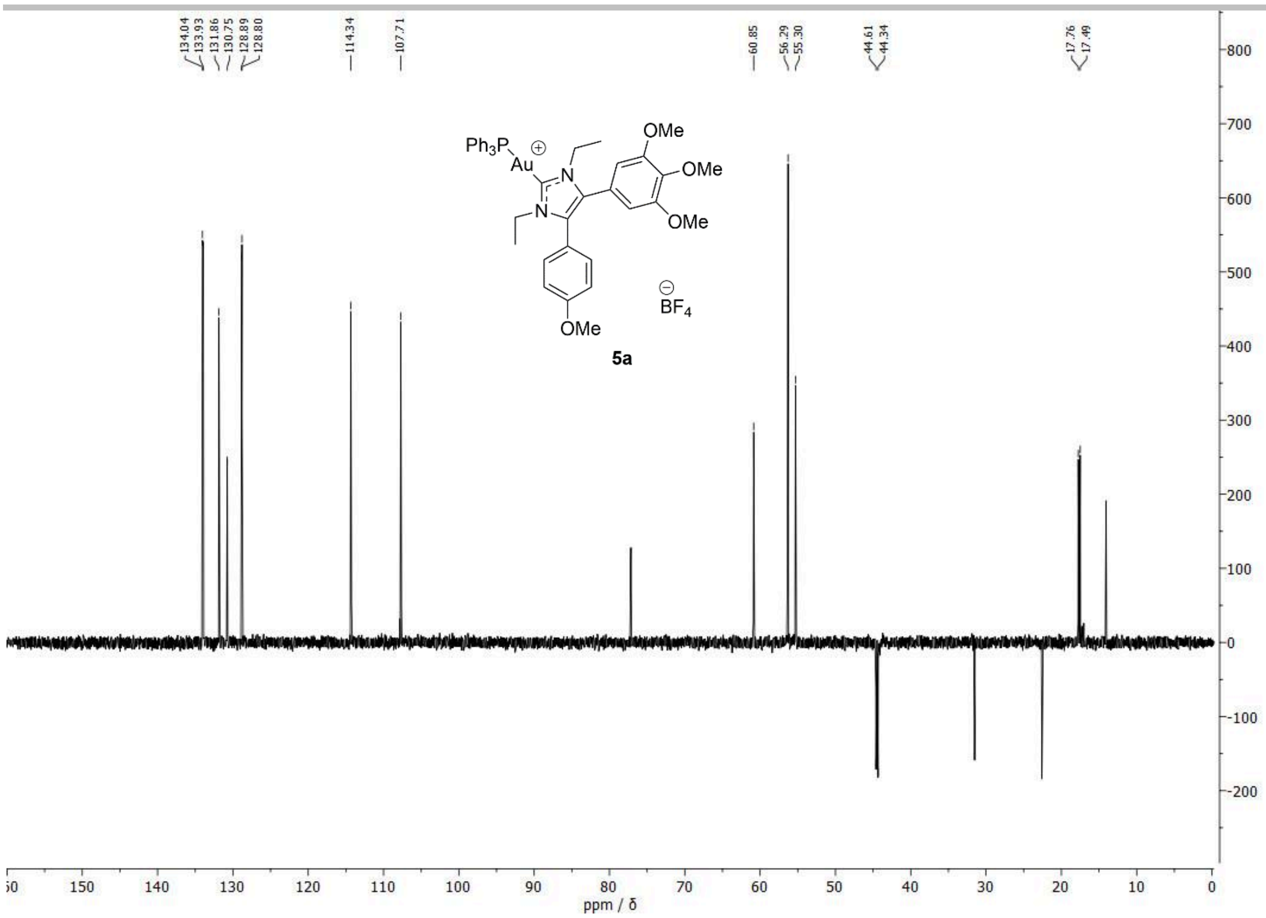


**Figure 4**: $^{13}C$ NMR (125 MHz, $CDCl_3$) spectrum of **5a.**

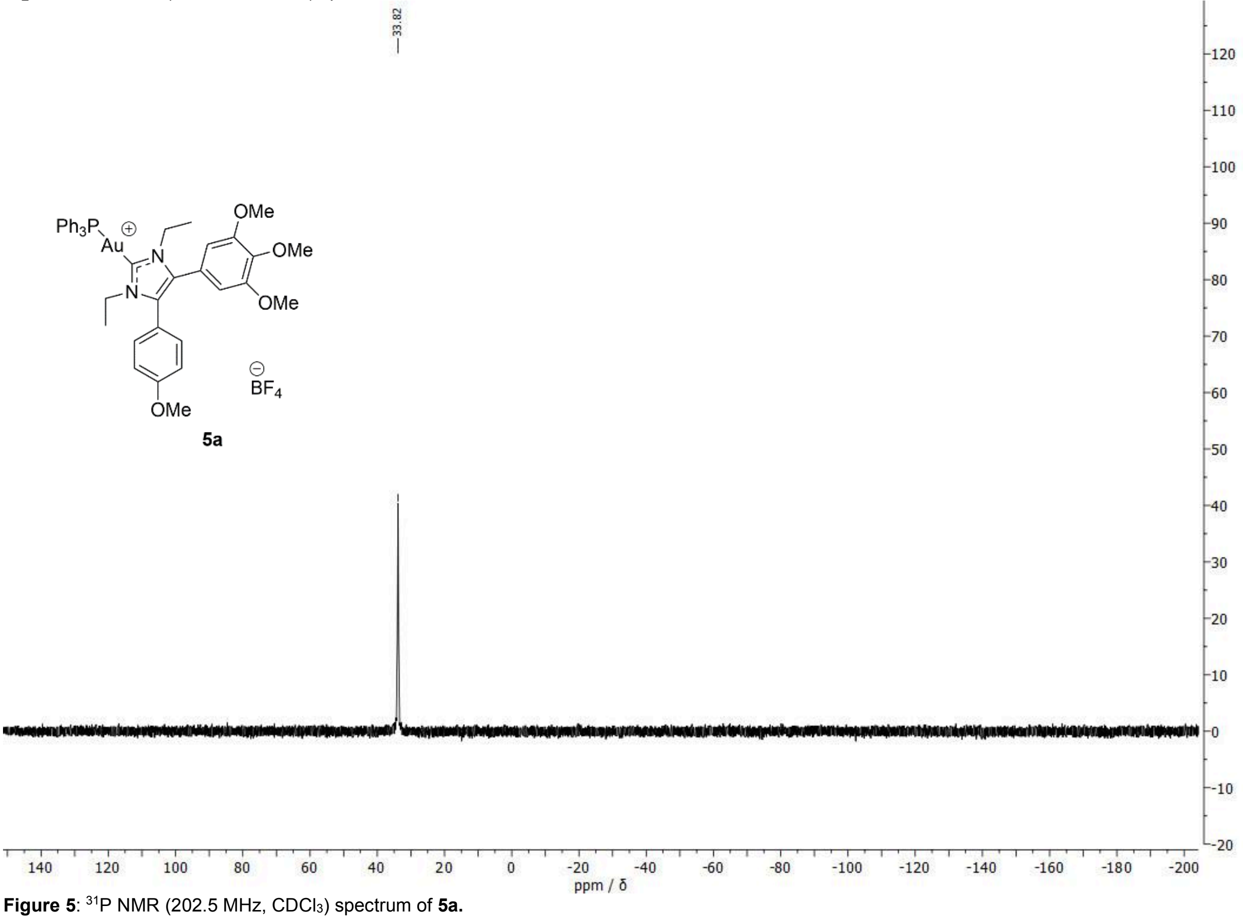


**Figure 5**: $^{31}P$ NMR (202.5 MHz, $CDCl_3$) spectrum of **5a.**

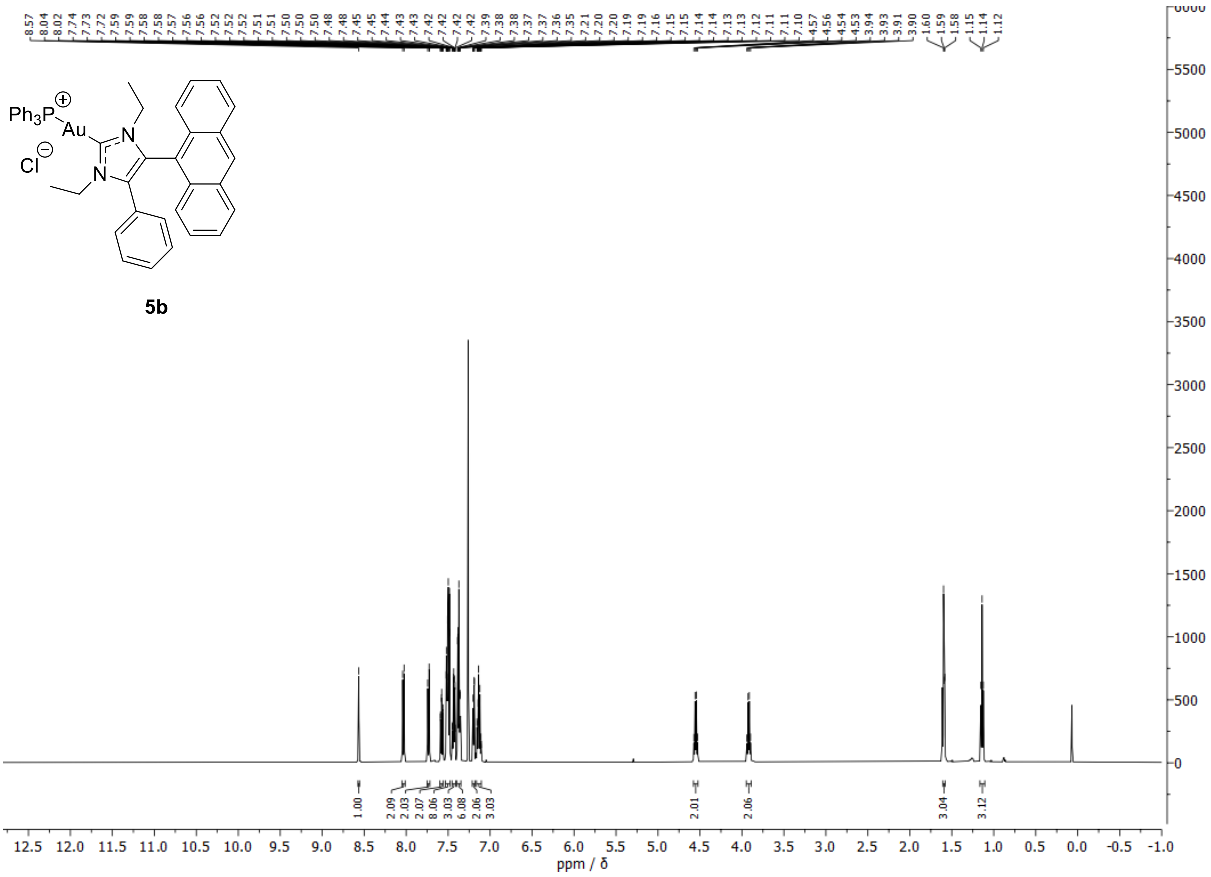


**Figure 6**: $^{1}H$ NMR (500 MHz, $CDCl_3$) spectrum of **5b.**

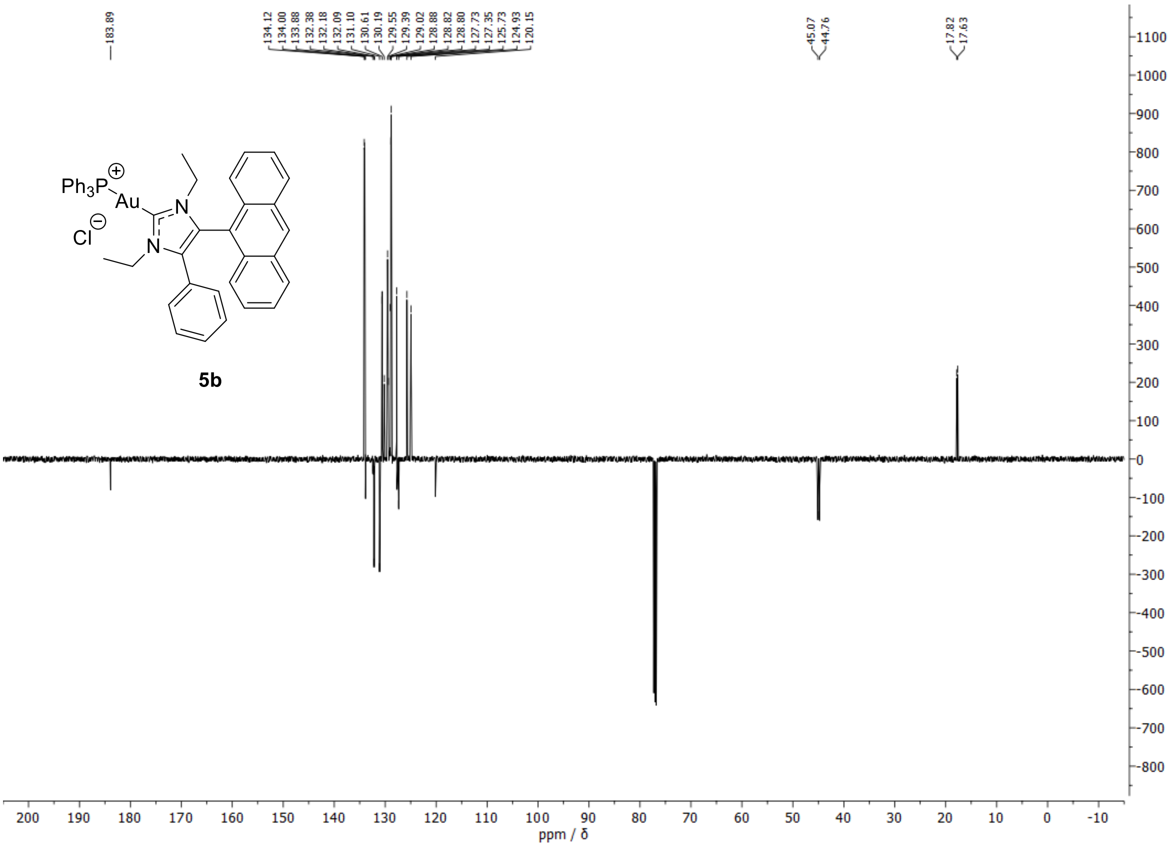


**Figure 7**: $^{13}C$ NMR (125 MHz, $CDCl_3$) spectrum of **5b.**

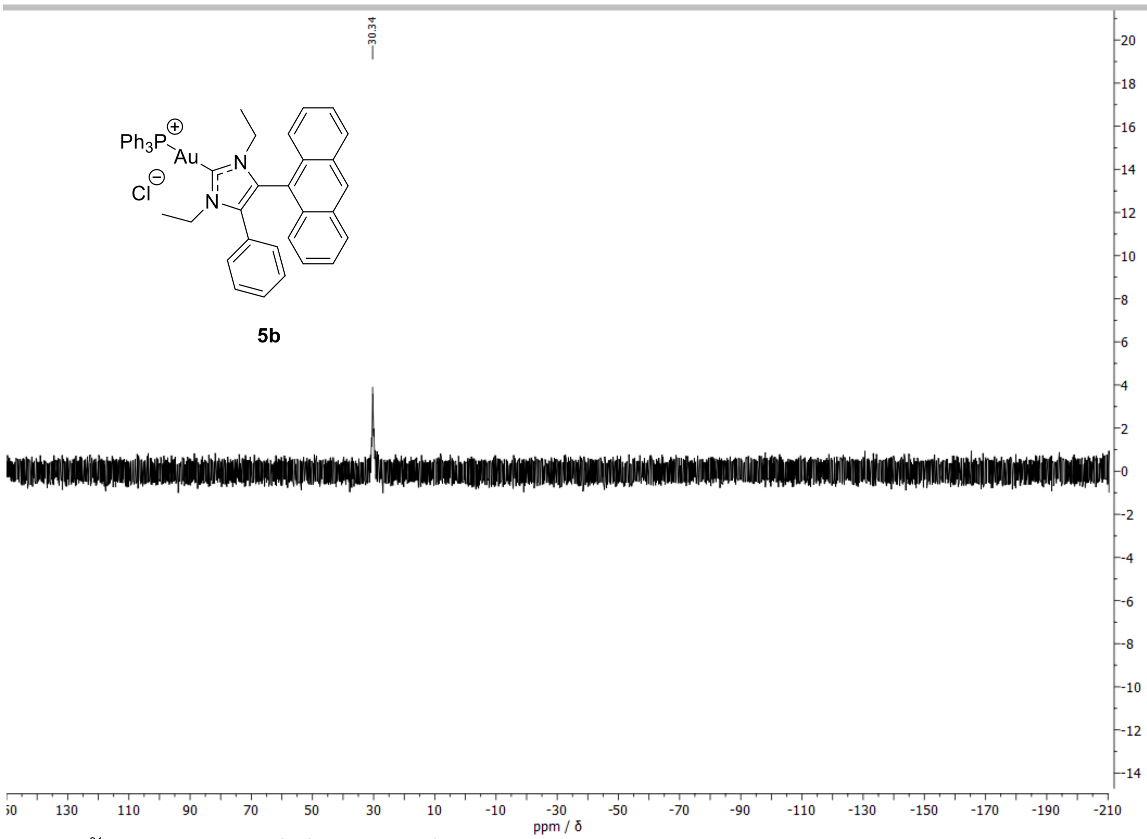


**Figure 8**: $^{31}$P NMR (202.5 MHz, $CDCl_3$) spectrum of **5b.**

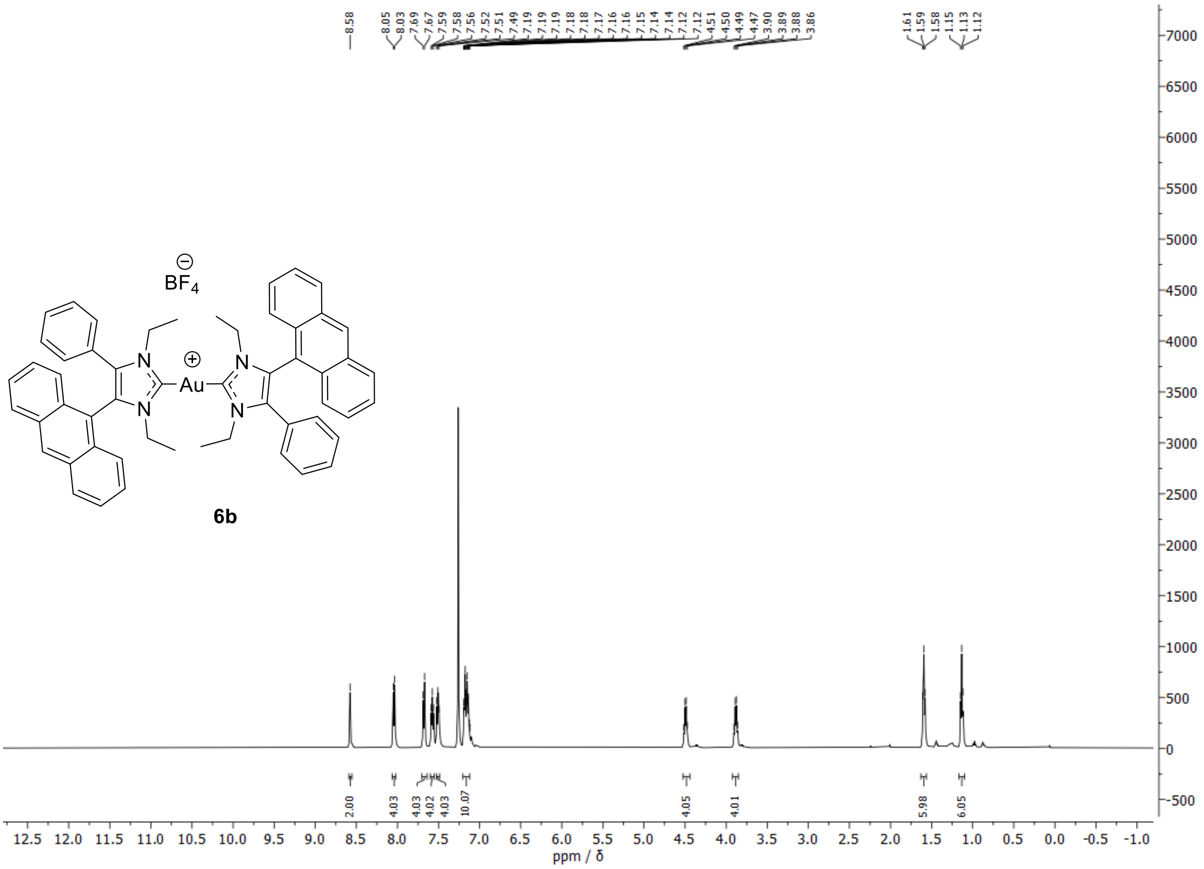


**Figure 9**: $^{1}$H NMR (500 MHz, $CDCl_3$) spectrum of **6b.**

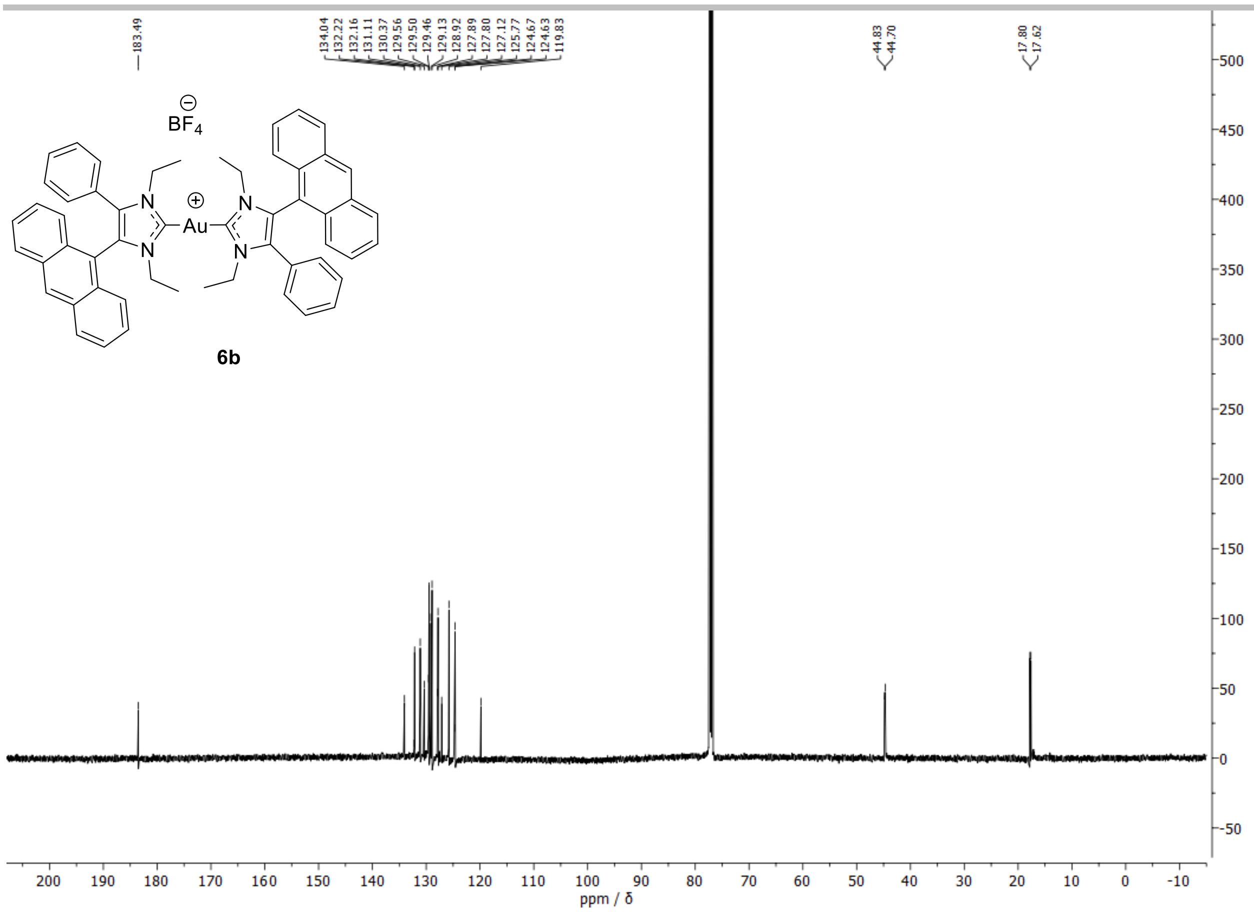


**Figure 10**: $^{13}C$ NMR (125 MHz, $CDCl_3$) spectrum of **6b.**

*Biochemical Evaluation*

Stock solutions of all test compounds were prepared (10 mM in DMF) and stored for at most one week at -20 °C. They were diluted, assay-depending, in dd$H_2O$, cell culture medium, or buffer.

**Cell lines and culture conditions.**

518A2 (Department of Radiotherapy and Radiobiology, University Hospital Vienna) human melanoma cells, HCT-116 (ACC-581) human colon carcinoma cells, as well as its p53 knockout mutant cell line HCT-116$^{p53-/-}$, HeLa cervix carcinoma, KB-V1$^{Vbl}$ multi-drug resistant cervix carcinoma cells, and HDFa (ATCC® PCS-201-012™) human dermal fibroblasts were grown in Dulbecco´s Modified Eagle Medium (DMEM; Biochrom) supplemented with 10% (v/v) fetal bovine serum (FBS; Biochrom) and 1% (v/v) Antibiotic-Antimycotic solution (Gibco). The cells were incubated at 37 °C, 5% $CO_2$, 95% humidified atmosphere and were serially passaged following trypsinisation by using 0.05% trypsin/0.02% EDTA (w/v; Biochrom GmbH, Berlin, Germany). The maximum-tolerated dose of vinblastine was added to the cell culture medium 24 h after every cell passage to keep the KB-V1$^{Vbl}$ cells resistant. The 518A2 melanoma cells are not available from cell banks, yet easily identified by their large size and flattened, spread-out morphology. Mycoplasma contamination was frequently monitored, and only mycoplasma-free cultures were used.

**Inhibition of cell growth (MTT assay).**[2]

The cytotoxic effect upon treatment with gold complexes **4**–**6** and auranofin for 72 h was determined by standard MTT assays. The tetrazolium salt 3-(4,5-dimethylthiazol-2-yl)-2,5-diphenyltetrazolium bromide (MTT; ABCR) is reduced by viable cells to a violet, water-soluble formazan. 518A2 melanoma cells, colon carcinoma cells HCT-116 and HCT-116$^{p53-/-}$, mdr KB-V1$^{Vbl}$ and HeLa cervix carcinoma cells ($5\times10^4$ cells $mL^{-1}$, 100 µL/well), as well as HDFa cells ($10\times10^4$ cells $mL^{-1}$, 100 µL/well) were seeded in 96-well tissue culture plates and cultured for 24 h at 37 °C, 5% $CO_2$ and 95% humidity. After treatment with the test compounds incubation of cells was continued for 72 h. Blank and solvent controls were treated identically. After addition of a 5 mg $mL^{-1}$ MTT stock solution in phosphate buffered saline (PBS), microplates were incubated for 2 h at 37 °C, centrifuged at 300 g, 4 °C for 5 min and the supernatant was discarded. The precipitate of formazan crystals was then redissolved in a 10% (w/v) solution of sodium dodecylsulfate (SDS; Carl Roth) in DMSO containing 0.6% (v/v) acetic acid. To ensure complete dissolution of the formazan, the microplates were incubated for at least 1 h in the dark. Finally the absorbance at $\lambda$ = 570 and 630 nm (background) was measured using a microplate reader (Tecan F200). All experiments were carried out in quadruplicate and the percentage of viable cells was calculated as the mean ± SD with controls set to 100%.

**Intracellular localisation of gold complexes.**
518A2 melanoma cells (500 µL/well, 0.5×10$^5$ cells/mL) were seeded on glass coverslips in 24-well plates and incubated under cell culture conditions for 24 h. The medium was aspirated and the cells were washed once with PBS. Nuclear counterstaining was performed with Nuclear Green LCS1 (5 µM; abcam) with an incubation time of 15 min under standard cell culture conditions. MitoTracker$^{TM}$ (Thermo Fisher) was used for mitochondrial staining, and the cells were incubated with this dye for 30 min. For lysosomal staining, the cells were incubated 20 min in an acridine orange solution (5 µg/mL, 400 µL/well). The cells were then washed twice with PBS and fresh DMEM was added. Then the cells were treated with the test compounds at a concentration of 30 µM and incubated for 30 min at standard cell culture conditions. The cells were fixed for 20 min at rt after a washing step with PBS in 3.7% formaldehyde solution in PBS, then washed three times and the coverslips were mounted in ProLong$^{TM}$ Gold Antifade Mountant (Invitrogen). The localisation of the test compounds was documented using confocal microscopy (Leica Confocal TCS SP5, 1000× magnification).

**Caspase-3/7 activation assay.**
For caspase activity measurements the Apo-ONE® Homogenous Caspase-3/7 Assay Kit (Promega Corp., Wisconsin, USA) was used. 518A2 melanoma cells (67.5 µL/well; 2×10$^5$ cells mL$^{-1}$) were grown in black 96-well plates for 24 h (37 °C, 5% $CO_2$ and 95% humidity). After incubation with different concentrations of the test compounds or solvent for 24 h under cell culture conditions, fluorogenic 1× caspase-3/7 substrate solution was added to each well and the substrate transformation by activated caspase-3/7 was performed for 45 min at rt. The fluorescence intensity ($\lambda_{ex}$: 485 ± 20 nm, $\lambda_{em}$: 530 ± 25 nm) was measured using a microplate reader (Tecan F200). Blank values (caspase-3/7 substrate solution plus test compound/solvent) were subtracted to reduce background signals, and a potential loss of cell viability after the incubation with the test compounds was taken into account by performing an MTT-assay as described above. The caspase-3/7 activity of the remaining vital cells was calculated as means ± SD with solvent controls set to 100%. All experiments were carried out at least in quadruplicate.

**Detection of morphological signs of apoptosis.**
518A2 melanoma cells (3 mL/well, 5×10$^4$ cells mL$^{-1}$) were grown in 6-well plates for 24 h (37 °C, 5% $CO_2$ and 95% humidity). After incubation with $IC_{50}$ concentrations of the test compounds (staurosporine: 500 nM, **4a**: 19.8 µM, **4b**: 7.9 µM, **5a**: 5.0 µM, **5b**: 2.9 µM, **6a**: 0.4 µM, **6b**: 5.5 µM) or solvent for 2.5 h under cell culture conditions, morphological changes of the cells were documented via brightfield microscopy (ZEISS Axiovert 135 and AxioVert MRc5, 100× magnification).

**Annexin-V-FITC/PI staining.**
For Annexin-V-FITC staining the TACS® Annexin-V-FITC Apoptosis Detection Kit (Trevigen, Maryland, USA) was used. 518A2 melanoma cells were seeded on glass coverslips (500 µL/well, 5×10$^4$ cells mL$^{-1}$) in 24-well plates, incubated under cell culture conditions (37 °C, 5% $CO_2$ and 95% humidity) for 24 h and treated with $IC_{50}$ concentrations of the test compounds (staurosporine: 500 nM, CDDP: 7.8 µM,[3] **4a**: 19.8 µM, **4b**: 7.9 µM, **5a**: 5.0 µM, **5b**: 2.9 µM, **6a**: 0.4 µM, **6b**: 5.5 µM) or solvent for a further 45 min under cell culture conditions. Afterwards the medium was aspirated and the cells were stained with 150 µL of Annexin-V-FITC/PI staining solution for 15 min according to the manufacturer´s instruction. Apoptosis-induced exposure of phosphatidylserine on the outer leaflet of the cytoplasmic membrane was documented by fluorescence microscopy (ZEISS Imager A1 AX10, 200× magnification). For differentiation between early apoptotic and late apoptotic/necrotic cells (which have lost membrane integrity), cells were counterstained with PI (propidium iodide).

**Ethidium bromide saturation assay.**
A potential DNA interaction of complexes **4**–**6** was assessed by a fluorescence-based ethidium bromide (EtdBr) staining assay. Salmon sperm DNA (SS-DNA, Sigma-Aldrich) in TE buffer (10 mM Tris-HCl, 1 mM EDTA, pH 8.5) was pipetted into a black 96-well plate to reach a final amount of 1 µg/100 µL assay volume and incubated with varying concentrations of test compounds for 2 h at 37 °C. Afterwards, 100 µL of EtdBr solution (10 µg mL$^{-1}$ in TE buffer) was added to each well. The fluorescence ($\lambda_{ex}$ = 535 nm, $\lambda_{em}$ = 595 nm) was measured using a microplate reader (Tecan F200) after 5 min of incubation. Each fluorescence value was corrected for intrinsic compound and EtdBr background fluorescence. A decreased fluorescence indicates an interaction between DNA and test compound which prevents the intercalation of EtdBr molecules into the double-stranded SS-DNA. All experiments were carried out in triplicate and the relative EtdBr fluorescence was quoted as means ± standard deviation with solvent controls set to 100%.

**Electrophoretic mobility shift assay (EMSA).**
Circular pBR322 plasmid DNA (1.5 µg; Thermoscientific) was incubated with dilution series (0, 25, 50, 75, 100 µM) of the test compounds or CDDP in TE-buffer (10 mM Tris-HCl, 1 mM EDTA, pH 8.5) for 24 h at 37 °C (20 µL total sample volume). Afterwards the DNA samples were subjected to DNA gel electrophoresis using 1% agarose gel in 0.5× TBE-buffer (89 mM Tris, 89 mM boric acid, 25 mM EDTA, pH 8.3). After staining the gels with an EtdBr solution (10 µg mL$^{-1}$ in 0.5× TBE-buffer) for 30 min, DNA bands were visualized using UV excitation. All experiments were performed at least in duplicate.

**Inhibition of thioredoxin reductase (TrxR) activity.**

For the measurement of thioredoxin reductase (TrxR) activity the TrxR Colorimetric Assay Kit (Cayman Chemical) was used according to manufacturer´s instructions. $1\times10^8$ 518A2 melanoma cells were harvested using a cell scraper, homogenised in 5 mL cold lysis buffer (50 mM $K_3PO_4$, 1 mM EDTA, pH 7.4) on ice and centrifuged for 15 min (4 °C, 10000×g). The protein concentration of the supernatant was determined via Bradford assays. Then, 10 µL Protease Inhibitor Cocktail Plus (Carl Roth) were added to 1 mL of the cell lysate which was either used for the assay right away or stored at -80 °C. Prior to use, the Assay Buffer was warmed to rt and the cell lysates, NADPH, aurothiomalate (ATM; specific TrxR inhibitor) and rat liver TrxR enzyme were thawed and kept on ice. After determination of the amount of cell lysate to use for optimum TrxR activity, all components were pipetted into the wells of a clear 96 well plate and the enzymatic reactions were initiated by addition of NADPH and 5,5´-dithio-bis(2-dinitrobenzoic acid) (DTNB). Then the absorbance at 405 nm was measured once every minute using a plate reader (Tecan F200) at at least ten time points. The TrxR activity was measured in the presence and absence of ATM. It is established that gold compounds such as ATM are highly specific inhibitors of mitochondrial TrxR.[4] Therefore, in presence of ATM an inhibition of TrxR can be assumed which allows a correction for TrxR-independent DTNB reduction (e.g. via glutathione). The difference between the two results renders the DTNB reduction due to TrxR activity. By plotting the average absorbance values as a function of time the slope of the linear portion of the curve was obtained, and the change of absorbance ($\Delta A_{405}$) per minute could be determined. The values were corrected for unspecific DTNB reduction and the TrxR activity was calculated using the following formula: TrxR activity [µmol/min/mL] = [corrected ΔA/min (sample) / 7.92 $mM^{-1}$] × [0.2 mL / 0.02 mL] × sample dilution. The assay was conducted at 22 °C. All experiments were performed in triplicate and the solvent-treated negative controls were set to 100%.

**Mitochondrial membrane potential.**

518A2 melanoma cells (100 µL/well, $0.25\times10^6$ cells/mL) were seeded in transparent (for viability control via MTT) and black 96-well plates, followed by an incubation period of 24 h under cell culture conditions. The medium was replaced by 90 µL/well standard assay buffer (80 mM NaCl, 75 mM KCl, 25 mM D-Glucose, 25 mM HEPES, pH 7.4 in $ddH_2O$) and the cells were treated with a volume of 10 µL of various concentrations of test compounds or solvent (DMF). CCCP (10 µM) served as a positive control. The cells were incubated for a further 45 min under standard cell culture conditions. Then 10 µL/well of a TMRM (tetramethylrhodamine methyl ester; Cayman Chemicals) solution were added (2 µM in standard assay buffer), followed by an incubation period of 10 min under exclusion of light. The cells were washed three times (160 µL PBS per well) and the fluorescence signal was measured after adding 100 µL PBS per well (Tecan F200, $\lambda_{ex}/\lambda_{em}$: 535/590 nm). The fluorescence signal was correlated to viability, determined by corresponding MTT assays.

**Determination of intracellular concentration of reactive oxygen species (DCFH-DA assay).**

518A2 melanoma cells were seeded in black 96 well plates (100 µL/well, $0.1\times10^6$ cells/mL) and incubated for 24 h under standard cell culture conditions. The medium was replaced by serum-free medium containing 20 µM DCFH-DA, followed by a further incubation periode of 30 min. Cells were washed twice with PBS (100 µL/well) and fresh medium without FBS was added (100 µL/well). After treatment with 10 µM of the test compounds or solvent, the cells were incubated for 1 h under standard cell culture conditions and subsequently washed twice with PBS. The cells were kept in PBS and the fluorescence was measured (Tecan F200, $\lambda_{ex}/\lambda_{em}$: 485/535 nm). Solvent-treated cells were taken as negative controls and their fluorescence was set to 100%.

**Lysosomal integrity.**

518A2 melanoma cells (500 µL/well, $0.05\times10^6$ cells/mL) were seeded on glass coverslips in 24 well plates and incubated for 24 h under standard cell culture conditions. Then the cells were treated with the test compounds at $IC_{50}$ concentrations and incubated for 1, 2, 4 or 6 h under standard cell culture conditions. 30 min before each time interval ended, cells were stained with Lysosomal Staining Kit Orange - Cytopainter (Abcam). To this end, medium was aspirated and the cells were washed once with 1 mL HHBS (Hanks Buffer with HEPES; 140 mM NaCl, 5 mM KCl, 1 mM $CaCl_2$, 0.4 mM $MgSO_4 \times 7H_2O$, 0.5 mM $MgCl_2 \times 6H_2O$, 0.3 mM $Na_2HPO_4 \times 2H_2O$, 0.4 mM $KH_2PO_4$, 6 mM D-glucose, 20 mM HEPES, pH 7.0). The cells were treated with 300 µL staining solution, incubated for 30 min under standard cell culture conditions, washed twice with 1 mL HHBS and fixed for 10 min at rt in 1 mL/well 3.7% formaldehyde in PBS. After three further washing steps, the coverslips were washed with 500 µL $ddH_20$ and embedded on glass slides with ProLong Gold™ (Invitrogen) containing 1 µg/mL DAPI. Lysosomal and nuclear staining was documented using a fluorescence microscope (Zeiss Imager A1 AX10, 400-fold magnification).

**Stability testing via NMR spectroscopy.**

Solutions of the test compounds were freshly prepared corresponding to stock solutions in dimethylformamide-$d_7$, 5 vol-% water-$d_2$ were added. $^1H$ NMR (500 MHz) spectra (16 scans) were measured at 0 h, 24 h, 48 h and 72 h to demonstrate the stability of the complexes in solution in the presence of water.

**Tubulin polymerisation assay.**

Purified porcine brain tubulin protein [5 mg/mL in Brinkley´s buffer 80 (BRB80)] containing 10% glycerol and 1.5 mM guanosine 5´-triphosphate (GTP)] was pipetted in a black 96-well half-area plate and mixed with the test compounds or solvent (DMSO) to a final concentration of 10 µM. The microplate was immediately placed in the pre-heated microplate reader (Tecan F200) and polymerisation was measured turbidimetrically at 37 °C by recording the absorption at 340 nm for 2 h in intervals of 20 s. All experiments were at least carried out in duplicate.

**Cell cycle analysis.**

518A2 melanoma cells (3 mL/well; 5×$10^4$ cells $mL^{-1}$) were grown on 6-well tissue culture plates for 24 h (37 °C, 5% $CO_2$, 95% humidity) and treated with different concentrations of the test compounds or solvent for another 24 h (37 °C, 5% $CO_2$, 95% humidity). The cells were harvested by trypsinisation and fixed in ice-cold 70% EtOH (1 h, 4 °C). After RNA digestion and propidium iodide (PI; Carl Roth) staining with PI staining solution (50 µg $mL^{-1}$ PI, 0.1% sodium citrate, 50 µg $mL^{-1}$ RNAse A in PBS) for 30 min at 37 °C to quantitatively stain DNA, the fluorescence intensity of 10 000 single cells was measured at $\lambda_{em}$ = 570 nm ($\lambda_{ex}$ = 488 nm laser source) with a Beckmann Coulter Cytomics FC500 flow cytometer. The percentage of cells in the different phases of the cell cycle (G1, S and G2/M phase) was determined by CXP software (Beckmann Coulter). The percentage of apoptotic and necrotic cells was derived from sub-G1 peaks.

## Results

**Influence on cellular morphology**
To confirm induction of apoptosis after treatment of 518A2 melanoma cells with complexes **4-6** we additionally documented morphological alterations via brightfield microscopy (Fig. 11).

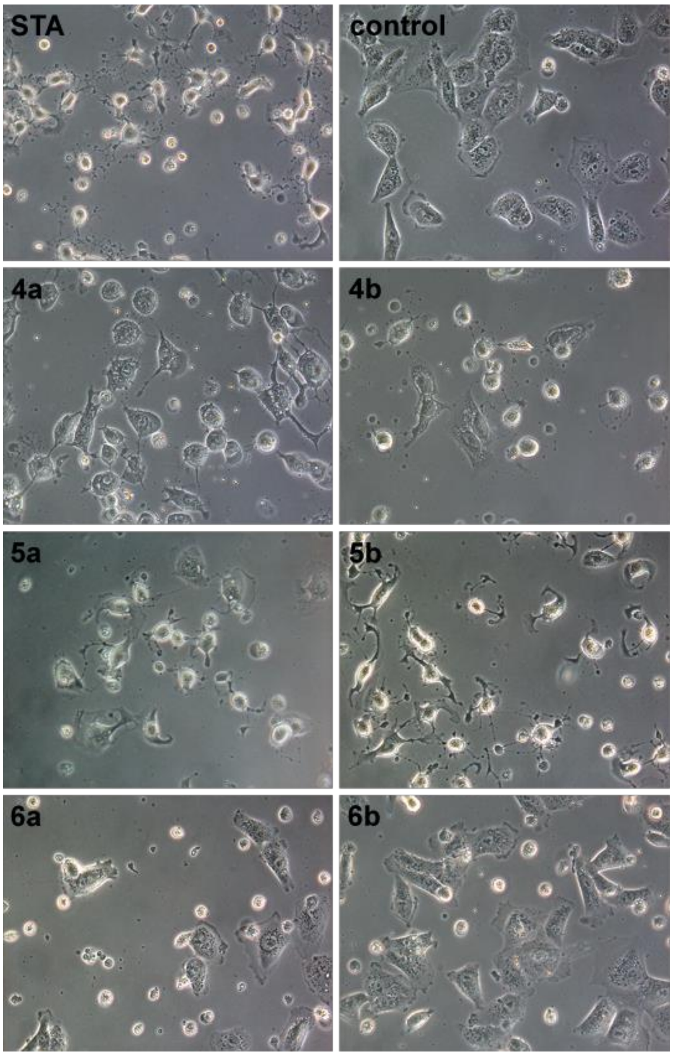


**Figure 11:** Morphological signs of apoptosis of 518A2 melanoma cells after incubation with staurosporine (STA; 500 nM) as well as $IC_{50}$ concentrations of gold complexes **4-6** for 2.5 h. (**4a**: 19.8 µM, **4b**: 7.9 µM, **5a**: 5.0 µM, **5b**: 2.9 µM, **6a:** 0.4 µM, **6b**: 5.5 µM). Documented using brightfield microscopy, 100× magnification. Images are representative of at least three independent experiments.

**Apoptosis detection using Annexin V-FITC and PI**

Early apoptotic events upon treatment of 518A2 melanoma cells with gold complexes **4-6** were detected by staining of phosphatitylserines on the outer surface of the cytoplasmic membrane with Annexin-V-FITC. Early apoptotic and late apoptotic/necrotic cells could be differentiated by counterstaining with propidium iodide (PI), which can only enter cells lacking membrane integrity. Results were documented via fluorescence microscopy (Fig. 12).

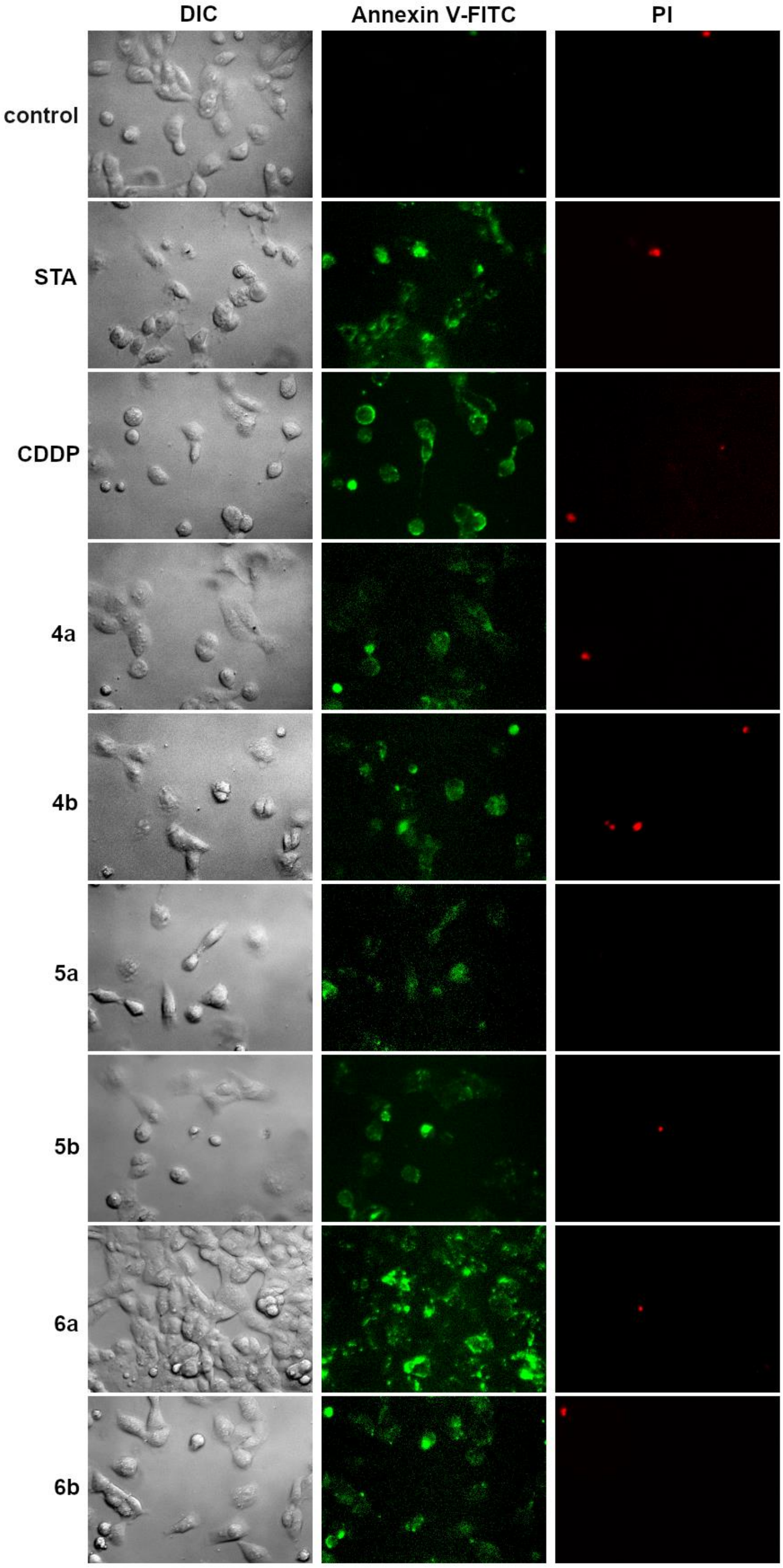


**Figure 12:** Annexin-V-FITC/PI staining of 518A2 melanoma cells treated with staurosporine (STA; 500 nM), CDDP (7.8 µM) as well as $IC_{50}$ concentrations of gold complexes **4-6** for 45 min. (**4a**: 19.8 µM, **4b**: 7.9 µM, **5a**: 5.0 µM, **5b**: 2.9 µM, **6a**: 0.4 µM, **6b**: 5.5 µM). Documented using fluorescence microscopy, 200× magnification. Images are representative for at least three independent experiments.

**Stability testing via NMR spectroscopy**

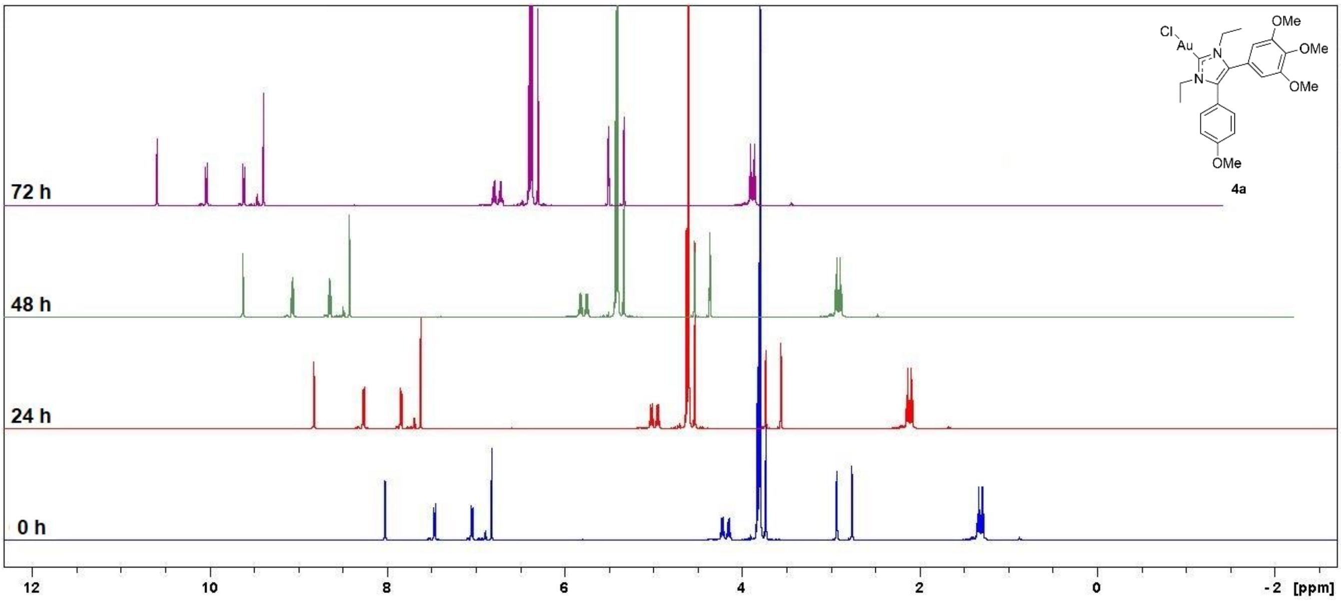


**Figure 13**: $^{1}$H NMR (500 MHz, dimethylformamide-d$_7$, 5 vol-% water-d$_2$) spectra of **4a**; 0 h, 24 h, 48 h and 72 h after preparing of stock solution.

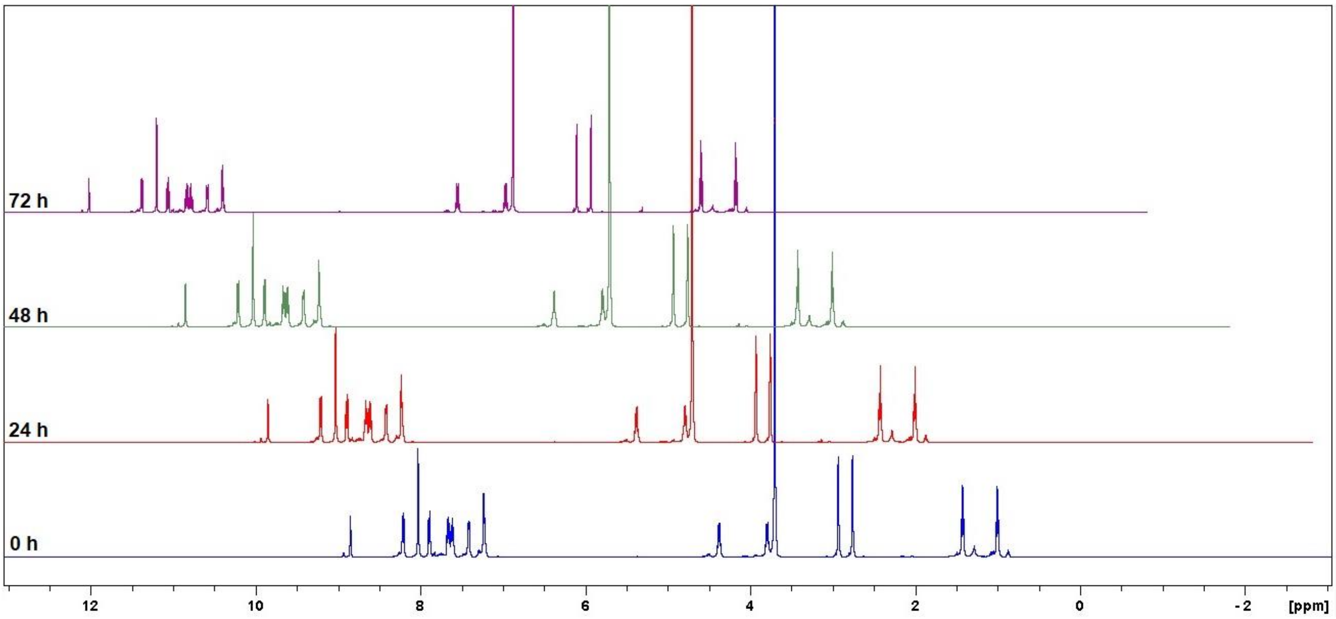


**Figure 14**: $^{1}$H NMR (500 MHz, dimethylformamide-d$_7$, 5 vol-% water-d$_2$) spectra of **4b**; 0 h, 24 h, 48 h and 72 h after preparing of stock solution.

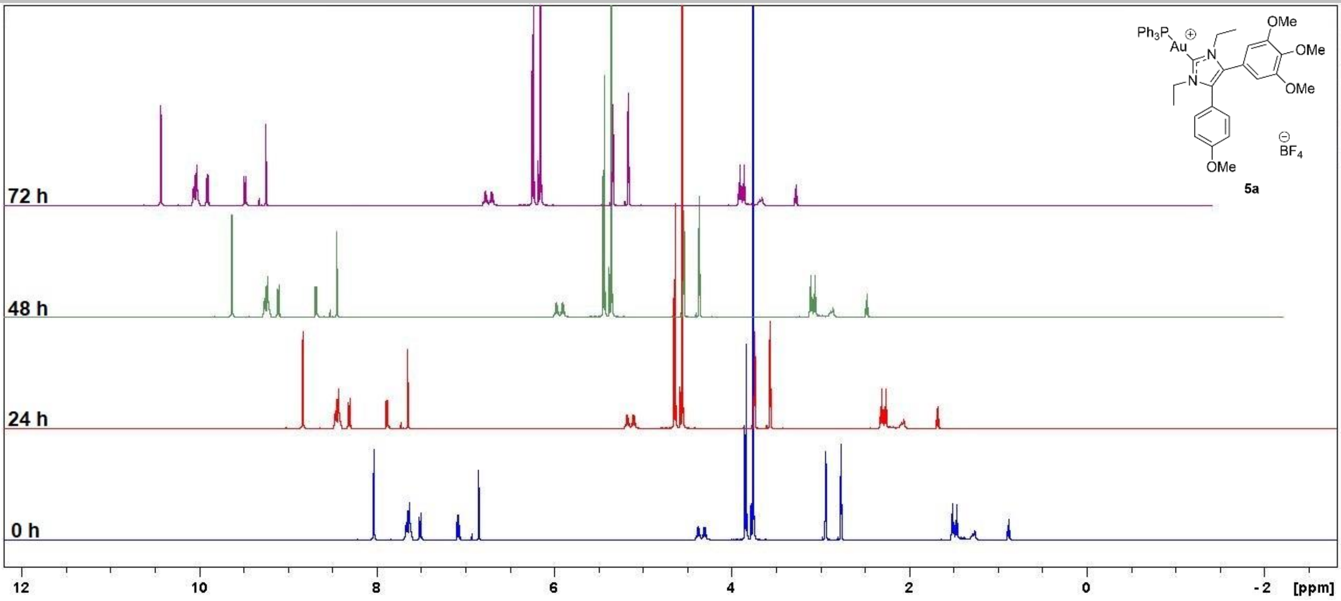


**Figure 15**: $^{1}$H NMR (500 MHz, dimethylformamide-d$_7$, 5 vol-% water-d$_2$) spectra of **5a**; 0 h, 24 h, 48 h and 72 h after preparing of stock solution.

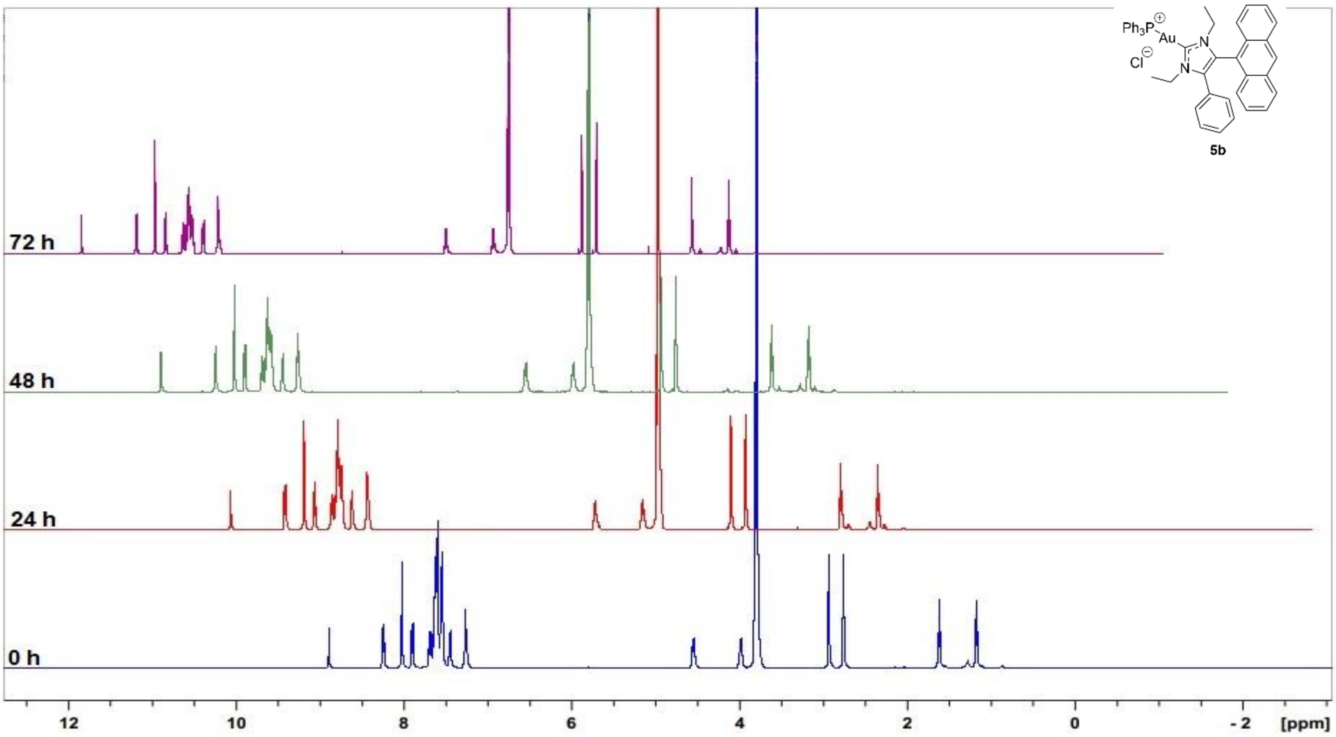


**Figure 16**: $^{1}$H NMR (500 MHz, dimethylformamide-d$_7$, 5 vol-% water-d$_2$) spectra of **5b**; 0 h, 24 h, 48 h and 72 h after preparing of stock solution.

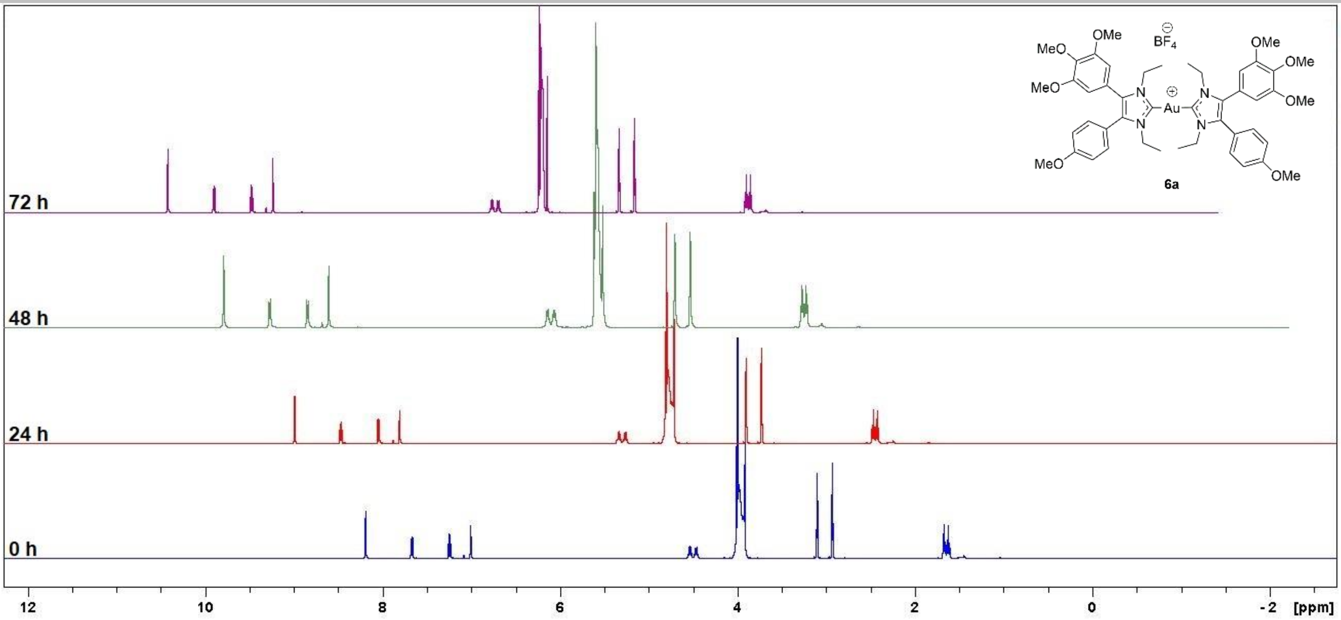


**Figure 17**: $^1$H NMR (500 MHz, dimethylformamide-$d_7$, 5 vol-% water-$d_2$) spectra of **6a**; 0 h, 24 h, 48 h and 72 h after preparing of stock solution.

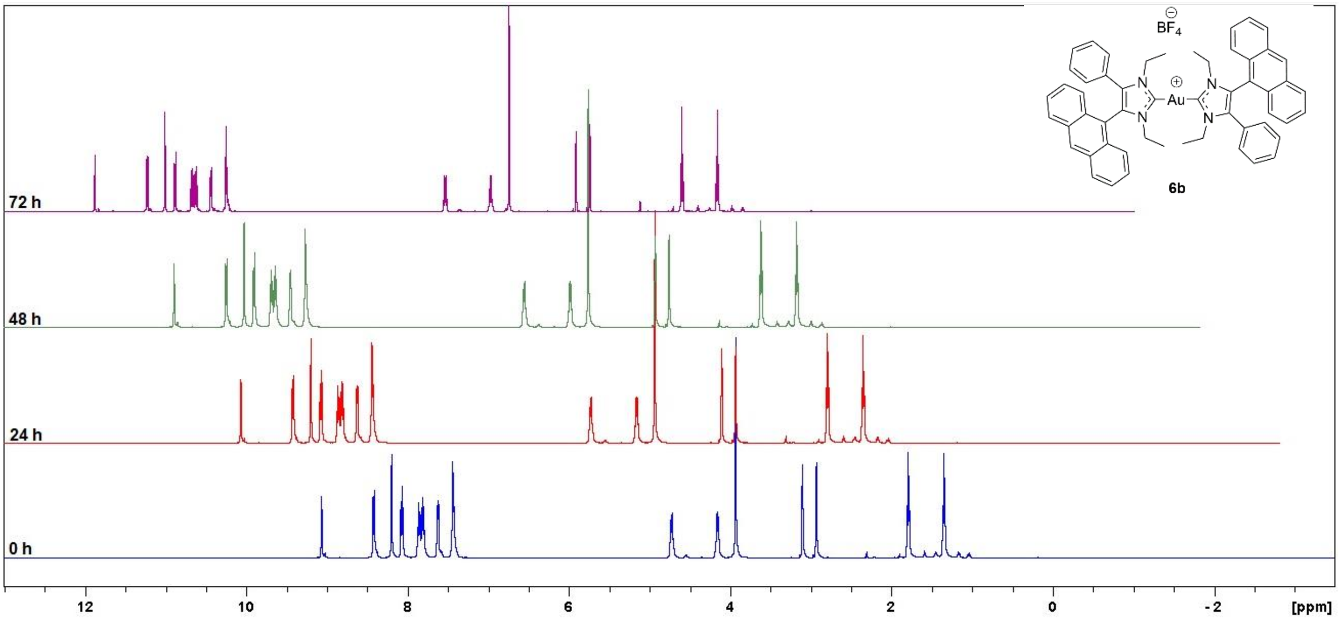


**Figure 18**: $^1$H NMR (500 MHz, dimethylformamide-$d_7$, 5 vol-% water-$d_2$) spectra of **6b**; 0 h, 24 h, 48 h and 72 h after preparing of stock solution.

**Interaction with tubulin**

Figure 19 shows the results from tubulin polymerization assays with complexes **4-6**. The known microtubule-destabilizing compound combretastatin A-4 (CA-4) was used as a positive control. With the exception of complexes **5**, which induce a slight inhibition of tubulin polymerization, no effects could be observed for the other test compounds **4** and **6**.

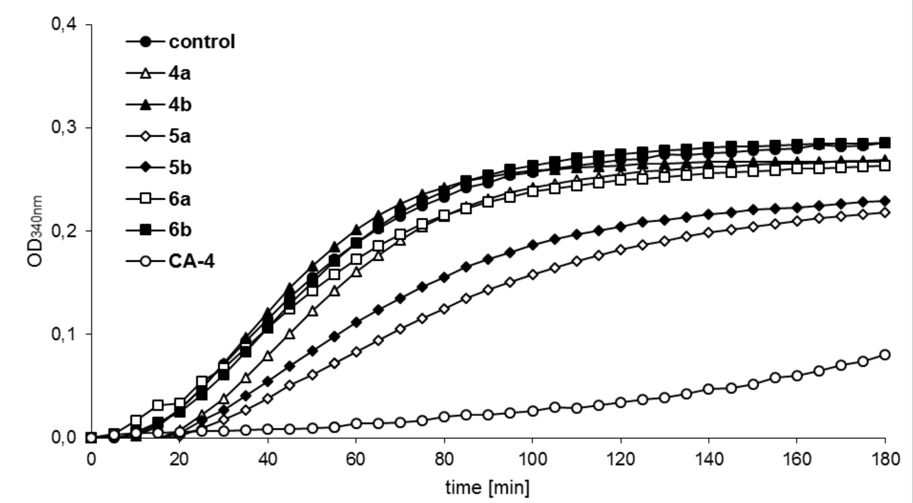


**Figure 19**: Turbidimetric measurement (OD at 340 ± 20 nm) of the polymerization of monomeric tubulin under the influence of the test compounds **4-6** at a final concentration of 10 µM. DMF was used as negative control, CA-4 at 10 µM was used as positive control. Values are representative of at least two independent measurements.

**Influence on the cell cycle of 518A2 melanoma cells**

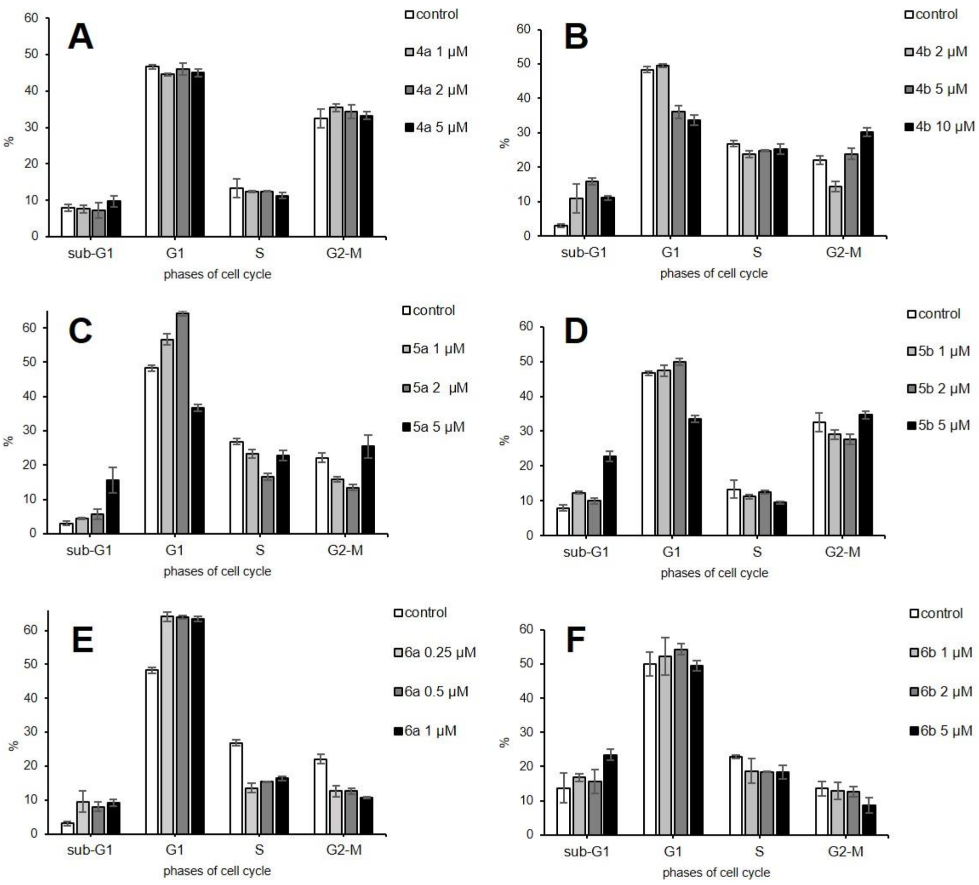


**Figure 20.** Influence of different concentrations of the test compounds **4a** (A), **4b** (B), **5a** (C), **5b** (D), **6a** (E) and **6b** (F) on the cell cycle of 518A2 melanoma cells measured via flow cytometry and PI staining; as a control the respective volume of solvent was used. Concentrations were adjusted to the $IC_{50}$ values of the compounds for 518A2 melanoma cells in MTT assays. Values are means ± standard deviation derived from three independent assays.

## References

[1] J. K. Muenzner, B. Biersack, H. Kalie, I. C. Andronache, L. Kaps, *et al.*, *Chem. Med. Chem.* **2014**, *9*, 1195-1204.
[2] T. Mosmann, *J. Immunol. Methods* **1983**, *65*, 55-63.
[3] M. Gold, Y. Mujahid, K. Ahmed, H. Kostrhunova, J. Kasparkova, *et al., J. Biol. Inorg. Chem*. **2019**, 24, 647-657.
[4] M. P. Rigobello, L. Messori, G. Marcon, M. A. Cinellu, M. Bragadin, *et al*, *J. Inorg. Biochem*. **2004**, 98, 1634-1641

## Author Contributions

**S. I. Bär:** Experimental studies and data acquisition (biochemical assays and stability tests), formal analysis, chemical analysis, project administration, writing of original draft, creation of graphics and art work; degree: lead, equal to M. Gold;
**M. Gold:** Experimental studies and data acquisition (biochemical assays and elemental analyses), formal analysis, project administration, writing of original draft, creation of graphics and art work; degree: lead, equal to S. I. Bär;
**S. W. Schleser:** Synthesis of gold(I) complexes, chemical analysis; degree: supporting;
**T. Rehm:** Synthesis of gold(I) complexes, chemical analyses; degree: supporting;
**A. Bär:** Synthesis of gold(I) complexes, writing of original draft; degree: supporting;
**B. Biersack:** Synthesis of gold(I) complexes, writing of original draft; degree: supporting;
**L. Köhler:** Experimental studies (EMSA); degree: supporting;
**L. R. Carnell:** Experimental studies (biochemical assays); degree: supporting;
**R. Schobert**: Project supervision, writing of original draft, funding acquisition, project coordination; degree: corresponding author;
